# MMP-2 Isoforms in Aortic Tissue and Serum of Patients with Ascending Aortic Aneurysms and Aortic Root Aneurysms

**DOI:** 10.1371/journal.pone.0164308

**Published:** 2016-11-01

**Authors:** Anke Tscheuschler, Philipp Meffert, Friedhelm Beyersdorf, Claudia Heilmann, Nadja Kocher, Xenia Uffelmann, Philipp Discher, Matthias Siepe, Fabian A. Kari

**Affiliations:** 1 Department of Cardiovascular Surgery, Heart Center, University of Freiburg, Freiburg, Germany; 2 Working Group on Molecular and Cellular Pathophysiology in Thoracic Aortic Disease, Dr. Barbara-Mez-Starck Cardiovascular Research Laboratory, Heart Center, University of Freiburg, Freiburg, Germany; Medical University Innsbruck, AUSTRIA

## Abstract

**Objective:**

The need for biological markers of aortic wall stress and risk of rupture or dissection of ascending aortic aneurysms is obvious. To date, wall stress cannot be related to a certain biological marker. We analyzed aortic tissue and serum for the presence of different MMP-2 isoforms to find a connection between serum and tissue MMP-2 and to evaluate the potential of different MMP-2 isoforms as markers of high wall stress.

**Methods:**

Serum and aortic tissue from n = 24 patients and serum from n = 19 healthy controls was analyzed by ELISA and gelatin zymography. 24 patients had ascending aortic aneurysms, 10 of them also had aortic root aneurysms. Three patients had normally functioning valves, 12 had regurgitation alone, eight had regurgitation and stenosis and one had only stenosis. Patients had bicuspid and tricuspid aortic valves (9/15). Serum samples were taken preoperatively, and the aortic wall specimen collected during surgical aortic repair.

**Results:**

Pro-MMP-2 was identified in all serum and tissue samples. Pro-MMP-2 was detected in all tissue and serum samples from patients with ascending aortic/aortic root aneurysms, irrespective of valve morphology or other clinical parameters and in serum from healthy controls. We also identified active MMP-2 in all tissue samples from patients with ascending aortic/aortic root aneurysms. None of the analyzed serum samples revealed signals relatable to active MMP-2. No correlation between aortic tissue total MMP-2 or tissue pro-MMP-2 or tissue active MMP-2 and serum MMP-2 was found and tissue MMP-2/pro-MMP-2/active MMP-2 did not correlate with aortic diameter. This evidence shows that pro-MMP-2 is the predominant MMP-2 species in serum of patients and healthy individuals and in aneurysmatic aortic tissue, irrespective of aortic valve configuration. Active MMP-2 species are either not released into systemic circulation or not detectable in serum. There is no reliable connection between aortic tissue—and serum MMP-2 isoforms, nor any indication that pro-MMP-2 functions as a common marker of high aortic wall stress.

## Introduction

A substantial proportion of cardiac deaths in western countries can be attributed to thoracic aortic aneurysm (TAA) rupture or dissection. The current indication to operate on TAAs has to rely exclusively on morphological criteria. Current guidelines suggest aortic repair at an aortic diameter of 45–50 mm, but recent studies suggest that this size criterion does not suffice to prevent dissection in many patients who suffer aortic complications at smaller diameters [1]. As a consequence, the need of additional radiological, biomechanical or biological markers of wall stress and risk of rupture or dissection is obvious.

Loss of elastic fibers and collagen degradation due to defective Extracellular Matrix (ECM) remodeling seems to be a hallmark of progressing aortic dilatation and is thought to be caused by imbalanced Matrix Metalloproteinase (MMP) activation and inhibition [2] [3] [4] [5] [6] [7] [8] [9]. MMP´s can be activated by removal of the N-terminal pro-domain. They are inactivated via interaction with Tissue Inhibitors Of Matrix Metalloproteinases (TIMP`s) [10] [11] [3] [12].

The MMP enzyme family is classified by substrate specificity and comprises collagenases, gelatinases, stromelysins, matrilysins and membrane-type (MT-) MMP`s. MMP-2 is one of the gelatinases and is regulated by an exclusive and well described mechanism. The inactive proenzyme is expressed constitutively and becomes activated through N-terminal cleavage. This occurs via pro-MMP-2, MT1-MMP and TIMP-2 interaction on the cell surface. The proportion of pro-MMP-2, TIMP-2 and MT1-MMP is an important factor for the regulation of pro-MMP-2 activation and the expression of MMP-2 and TIMP-2 is connected to each other.[13] [14] [15] [10].

A mouse model recently revealed, that MMP-2 is involved in the synthesis and degradation of ECM structure proteins and that its function in ECM protein synthesis seems to play an important role in thoracic aortic aneurysms especially. [16].Moreover, mechanical stretch, which is one form of mechanical stress, has been found to stimulate pro-MMP-2 release in vascular smooth muscle cells (VSMC`s) [17]. MMP-2 protein levels were observed to be higher in aortic regions with enhanced shear stress[18] and adaptive responses to high wall shear stress have been shown to be connected to pro—and activeMMP-2 in an animal mode l[19]. Wall shear stress in ascending aortic aneurysms was shown to increase with aortic diameter [20] and blood flow patterns in ascending aortic aneurysms seem to be associated with aortic morphology [21]. Taken together, these findings suggest that MMP-2 availability plays an important role in aortic wall pathology and that MMP-2 isoforms may serve as indicators of mechanical stress in the aortic wall. Therefore, we aimed to evaluate the potential of MMP-2 isoforms as markers of high wall stress by analyzing patient serum and aortic tissue for the existence of pro- and active MMP-2.

## Patients and Methods

### Patients

N = 24 patients with proximal thoracic aortic aneurysms of the aortic root and/or ascending aorta (>45mm) were included in this study. Inclusion criterion was age 18 to 75 years, no selection was made regarding bicuspid or tricuspid aortic valves, valve function and origin of the aneurysm. 1 patient had Marfan`s syndrome, 1 patienthad ACTA2 mutation. Exclusion criteria were active endocarditis, active malignancy, children / octogenarians, and acute aortic dissection.

24 patients had ascending aortic aneurysms, 10 of them also had also aortic root aneurysms. Three patients presented normally functioning valves, 12 had regurgitation alone, eight had regurgitation and stenosis and one had only stenosis. For further details on clinical variables see Table 1.

**Table 1:** Patient characteristics.

Factor	Group	Overall
n		24
Age (mean (SD))		58.0 (14.9)
Gender (%)	Female	4 (16.7)
	Male	20 (83.3)
Hyperlipidemia (%)		12 (50.0)
Hypertension (%)		14 (58.3)
BMI (%)	<25	6 (25.0)
	≥25	18 (75.0)
BAV (%)		9 (41.7)
Asc.Aor.Dia (mean (SD))		53.06 (9.1)
Medication		
ACE.I (%)		8 (33.3)
Anticoagulant (%)		8 (33.3)
beta.Blocker (%)		10 (41.7)
Sartane (%)		3 (12.5)
MMP-2 [ng/ml](mean(SD))		190.2 (46.4)

BMI, body mass index; BAV, bicuspid aortic valve; ACE.I, angiotensin-converting enzyme inhibitor; SD, standard deviation; Asc.Aor.Dia, ascending aortic diameter.

This study was approved by the Ethics Committee of the University of Freiburg and informed written consent was obtained from all patients. Serum from n = 19 healthy individuals was studied as control (Table 2).

**Table 2:** Characteristics of serum control group.

Factor	
N	19
Age (mean (SD))	29.2 (6.2)
Gender (%)	• F 9 (37.4) • M 10 (52.6)
MMP-2 [ng/ml] (mean (SD))	240.3 (46.4)

Female, F; Male, M; SD, Standard Deviation

### Sample preparation and protein extraction

Blood samples were taken one day before surgery, centrifuged for 10 minutes at 1600 rpm, aliquoted and deep frozen at -80°C within 1 hour. Aortic tissue resected from the anterior part of the aortic wall was deep frozen in liquid nitrogen and stored at -80°C. To extract proteins, frozen aortic tissue including all layers of the aorta was pulverized under liquid nitrogen and supplied with 500 μl pre-cooled lysis buffer (50mM Tris, 150mM NaCl, 1% Triton-X-100, pH 7,5) per 100 mg freshweight. Samples were vortexed, kept on ice for 60 minutes and vortexed again every 15 minutes. Samples were centrifuged at 13 000 rpm and 4°C for 15 minutes. The supernatant was filtered through spin-x centrifuge filters (0,22 μm cellulose acetate, Costar) by centrifugation at 13000 rpm and 4°C for 15 minutes and kept on ice until further processing. The pellet was resuspended in 200 μl lysis buffer and kept on ice for another 30 minutes with vortexing every 15 minutes followed by another centrifugation at 13000 rpm and 4°C for 15 minutes. The supernatant was filtered and processed as described above. Both supernatants were combined and protein extracts were aliquoted and stored at -20°C. Total protein content of protein extracts and serum was determined by BCA assay following the manufacturer`s instructions (Thermo Scientific Pierce BCA Protein Assay).

### Enzyme-linked-Immunosorbent Assay

MMP-2 concentrations in serum samples were determined using quantitative sandwich enzyme-linked-immunosorbent-assay (MMP200, R&D Systems Europe) according to the manufacturer`s instructions. Each standard and sample was measured in duplicate.

### Gelatin zymography

Gelatin zymography was performed as described previously [22] with slight modifications. All sera, protein extracts and controls were diluted with zymography buffer (25mM Tris, 150mM NaCl, 10mM CaCl_2_, 0,2% Brij-35, pH 7,5) containing protease inhibitor (P8340, Sigma-Aldrich). A total protein amount of 15 μg per lane was loaded onto 8% SDS gels containing 0,2% gelatin (gelatin from porcine skin G1890, Sigma-Aldrich). Electrophoresis was conducted for 2,5 hours at 20 mA per minigel. Gels were washed twice with 2,5% Triton for 30 minutes at room temperature with agitation. Enzymatic digestion of gelatin occurred through incubation in zymography buffer at 37°C for 19 hours. The gels were then stained with 50 ml 0,2% Coomassie brilliant blueR-250 per gel (Serva electrophoresis GmbH) following the manufacturer`s instructions. MMP-2 was identified via a human full length MMP-2 protein (ab 168864, Abcam) isolated from rheumatoid synovial fibroblasts (Abcam technical service, personal communication) as a positive control.

Semiquantitative determination of MMP-2 isoforms was performed by analyzing pixel density with Image J. Each sample value was normalized to 0.33 ng human full length MMP-2 analyzed in each gel. As human full length MMP-2 always contained pro- and active MMP-2, pixel densities of both measured areas were summarized. Plotted values for aortic tissue pro–or active MMP-2 represent the mean of three independent experiments. Plotted total MMP-2 values were calculated by summarizing pro- and active MMP-2 means for each sample. Dilution series for zymography validation were carried out 3 times independently. Values represent the mean of two samples with the same dilution factor ran in the same gel. Calibration curves were run 4 times independently with 2 lanes containing the same MMP-2 amount in each gel.

### Western blot

Electrophoresis was conducted as described under gelatin zymography. Samples were diluted with protein extraction buffer and a total protein amount of 20 μg per lane was loaded. Proteins were blotted on PVDF membranes (Immobilon-P, Merck-Millipore) for 1 hour at 5.5 mA per minigel and 200 V with Towbin buffer [23] (Trans-Blot SD Semi-Dry Transfer Cell, Bio-Rad). Membranes were blocked for 30 minutes using 2% nonfat dry milk diluted in TBS-T. MMP-2 immunodetection occurred via incubation with MMP-2 primary antibody (MAB902, R&D Systems Europe; diluted 1:5000 in TBS-T) over night at 4°C. After threefold washing with TBS-T, blots were incubated with secondary antibody (HAF 109, R&D Systems Europe, diluted 1:20000 in TBS-T) for 1 hour at room temperature with agitation. After four fold washing with TBS-T, blots were developed using Pierce ECL-Prime Western Blotting Substrate following the manufacturer`s instructions.

### Activation of Pro-MMP-2

Pro-MMP-2 was converted into the active 65 kDa form by incubation with 2mM 4-aminophenylmercuric acetate (APMA, Sigma-Aldrich) for 2 hours at 37°C. Serum was diluted in zymography buffer to a total protein content of 2μg/μl prior to incubation with APMA. Protein extracts were incubated with APMA with and without pre-dilution in zymography buffer at a final total protein content of 2–4 μg/μl. Standards were activated following the manufacturer`s instructions.

### Statistical analyses

All statistical analyses were carried out with SigmaPlot 13 (Systat Software GmbH, Erkrath Germany). To identify differences between groups, a two-tailed t-test was used after testing for normal distribution by Shapiro- Wilk test. Numerical variables are given as means and standard deviations. For correlation analyses, Pearson correlation analysis was used after positive testing for normal distribution. For correlation analysis between MMP-2 and the aortic diameter, we used the Spearman correlation analysis as aortic diameters are not normally distributed.

### Image processing

**Fig 1:**
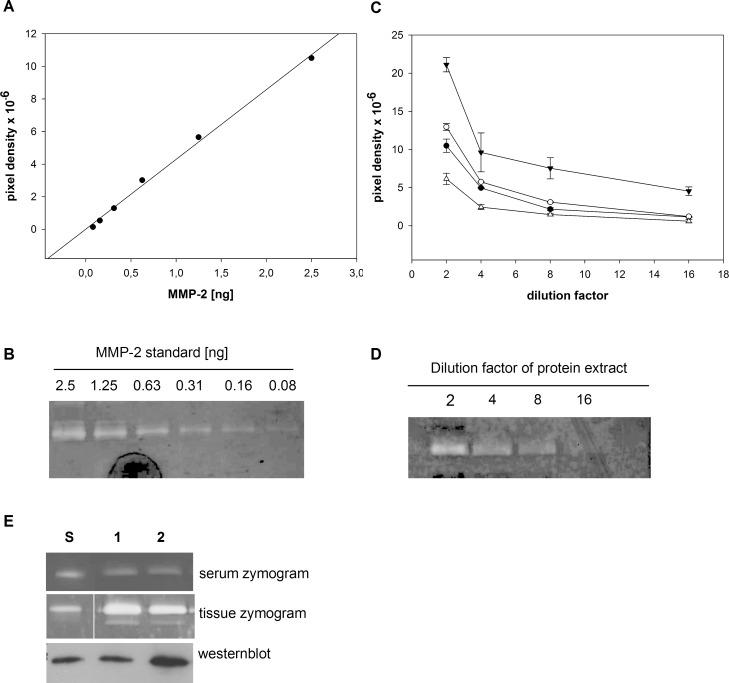
Validation of gelatin zymography. **A.** Dilution series of MMP-2 standard. A standard curve was generated from human full length MMP-2 from 0–2.5 ng of total MMP-2. Pixel density measured from zymograms showed a linear relationship with the MMP-2 amount. **B.** Representative zymogram of standard dilution series reveals decreasing signal strength in conjunction with declining MMP-2 amount. **C.** Dilution series of protein extracts gained from patients with ascending aortic aneurysms. Protein extracts were diluted from 2fold until 16fold. The measured pixel density in the zymograms showed a linear decrease at dilution factors between 2fold and 8fold. Measurements of 4 independent dilutions illustrated the assay`s reproducibility. Each value represents the mean of two samples at the same dilution measured in the same gel. **D.** Representative zymogram of protein extract dilution series shows decreasing signal strength in conjunction with an increasing dilution factor. **E.** Representative serum and tissue zymograms showing different gelatinolytic activities. serum zymogram shows gelatinolytic activities corresponding to pro-MMP-2. Tissue zymogram shows gelatinolytic activities corresponding to pro-MMP2 and active MMP-2. Westernblot confirmed that the detected bands were MMP-2. **1,2**: patient sample. **S:** Human full length MMP-2.

**Fig 2:**
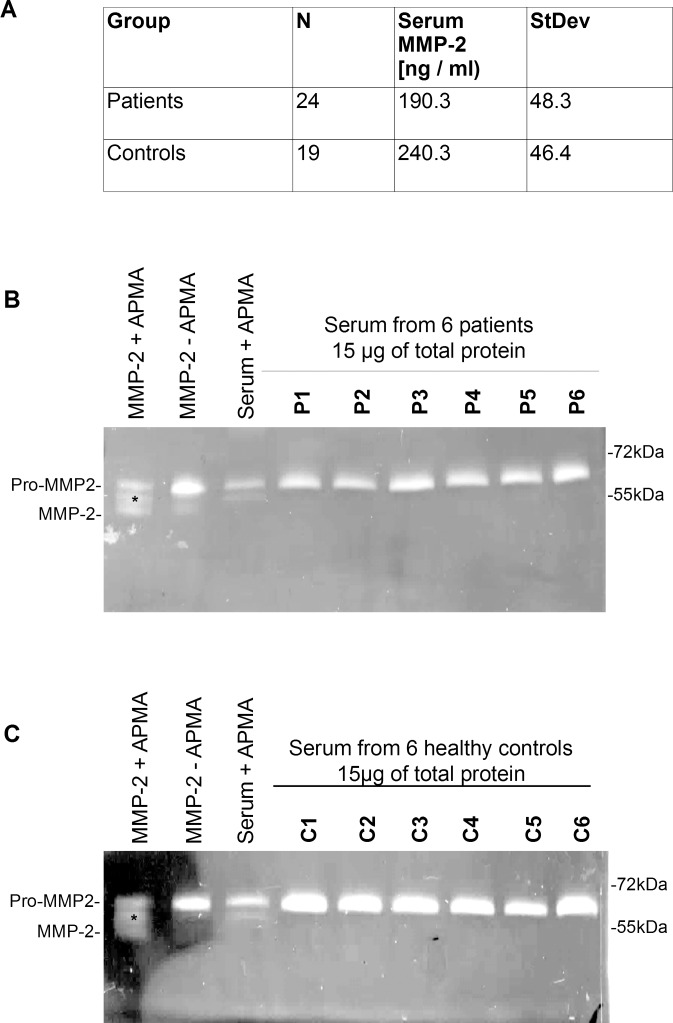
Serum MMP-2 in patients with ascending aortic aneurysms and healthy controls. **A.** Serum MMP-2 levels in patients and healthy controls measured by ELISA. Two-tailed t-test revealed a significant difference in serum MMP-2 levels in both groups (P = 0,00138). MMP-2 serum levels were lower in patients than in healthy controls (190.3 +/- 48.3 vs 240.3 +/- 46.4). **B.** Representative zymogram showing gelatinolytic activity in serum from 6 patients with ascending aortic aneurysms. Lane 1: Human full length MMP-2 activated with 2 mM APMA for 2 hours. Lane 2: Human full length MMP-2 as delivered. Lane 3: one representative serum sample activated with 2 mM APMA. Lane 4–9: serum from patients with ascending aortic aneurysms. Asterisk indicates 67 kDa intermediate MMP-2 isoform present in MMP-2 standard with and without APMA incubation and in serum incubated with APMA. Lanes with serum samples show gelatinolytic activity corresponding to pro-MMP-2 only. **C.** Zymogram showing gelatinolytic activity from 6 serum samples from healthy controls. Lane 1: Human full length MMP-2 activated with 2 mM APMA. Lane 2 Human full length MMP-2 as delivered. Lane3: one representative serum sample activated with 2 mM APMA Asterisk indicates 67 kDa intermediate MMP-2 isoform present in human full length MMP-2 with and without APMA incubation and in serum incubated with APMA. Lanes with serum samples show gelatinolytic activity corresponding to pro-MMP-2 only.

**Fig 3:**
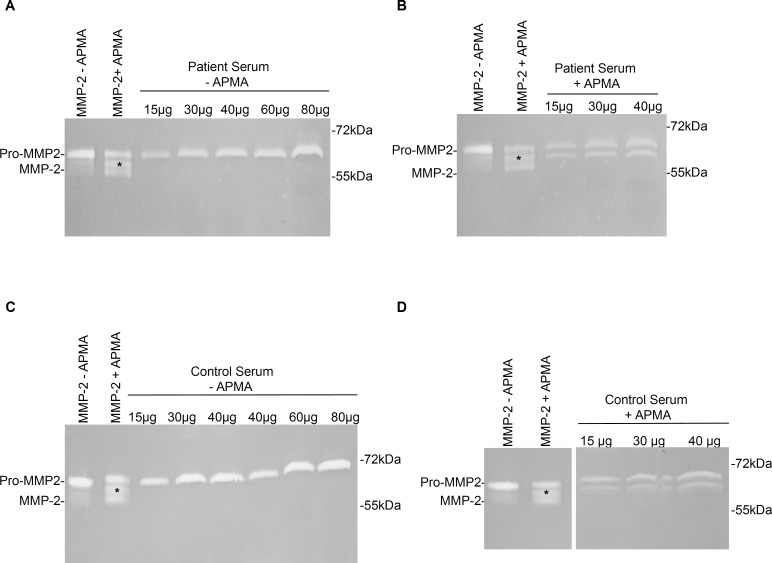
Representative zymograms showing gelatinolytic activity in serum from patients with ascending aortic/aortic root aneurysms and healthy controls. Increasing amounts of total protein were analyzed to detect possible signals close to detection limit. Lane 1: Human full length MMP-2 as delivered. Lane 2: Human full length MMP-2 activated with 2 mM APMA for 2 hours. Asterisk indicates 67 kDa intermediate MMP-2 isoform present in MMP-2 standard with and without APMA incubation and in serum incubated with APMA. **A.** Serum from patients with ascending aortic aneurysms was analyzed in total protein amounts from 15 μg to 80 μg. Whereas signals corresponding to pro-MMP-2 increased, no signals relatable to active MMP-2 became visible. **B**. Serum from patients with ascending aortic aneurysms was incubated with 2mM APMA for 2 hours at 37°C and analyzed in increasing amounts of total protein from 15 μg to 40 μg. Signals related to pro-MMP-2 and intermediate MMP-2 became stronger with increasing amount of total protein. But no signal relatable to the 65kDa active MMP-2 appeared. **C.** Serum from healthy controls was analyzed in total protein amounts of 15 μg until 80 μg. signals increased in the same way as described for the serum samples from patients with ascending aortic aneurysms. No signals corresponding to active MMP-2 appeared. **D.** Serum from healthy controls was analyzed in total protein amounts of 15 μg until 40 μg after incubation with APMA for 2 hours at 37°C. Signals for pro-MMP-2 and intermediate MMP-2 increased with protein amount but no signals relatable to active MMP-2 appeared.

**Fig 4:**
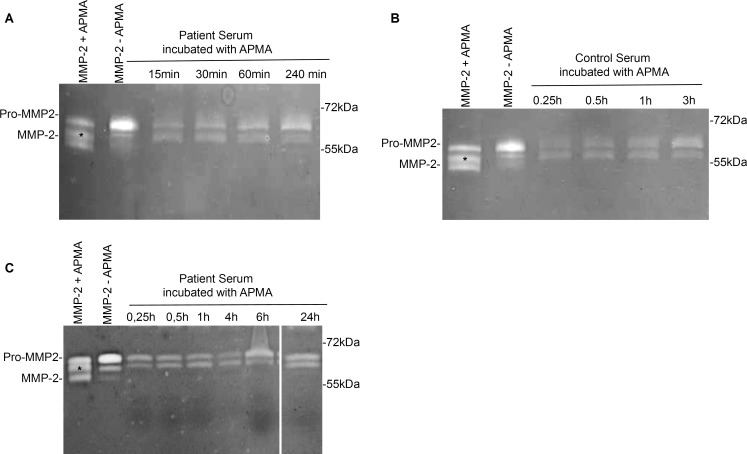
Representative zymograms revealing gelatinolytic activity in serum after incubation with APMA for different incubation times. Serum from patients with ascending aortic aneurysms and healthy controls was incubated with 2 mM APMA for different times to exclude that active MMP-2 can be generated from serum MMP-2 after longer incubation times. Lane 1: Human full length MMP-2 after activation with 2mM APMA for 2 hours. Lane 2: Human full length MMP-2 as delivered. **A.** Serum from patients with ascending aortic aneurysms was incubated with 2mM APMA for 15–240 minutes. All lanes show bands corresponding to pro-MMP-2 and bands corresponding to intermediate MMP-2. No signals for active 65kDa MMP-2 became visible after prolonged incubation time with APMA. **B.** Serum from healthy controls was incubated with 2mM APMA for 15–240 minutes. Lanes show bands corresponding to pro-MMP-2 and intermediate MMP-2. No signals relatable to active MMP-2 appeared after prolonged incubation times with APMA. **C.** Serum from patients with ascending aortic aneurysms was incubated with 2mM APMA for 0,25–24 hours. All lanes show bands corresponding to pro-MMP-2 and intermediate MMP-2. No signals relatable to active MMP-2 were detectable.

**Fig 5:**
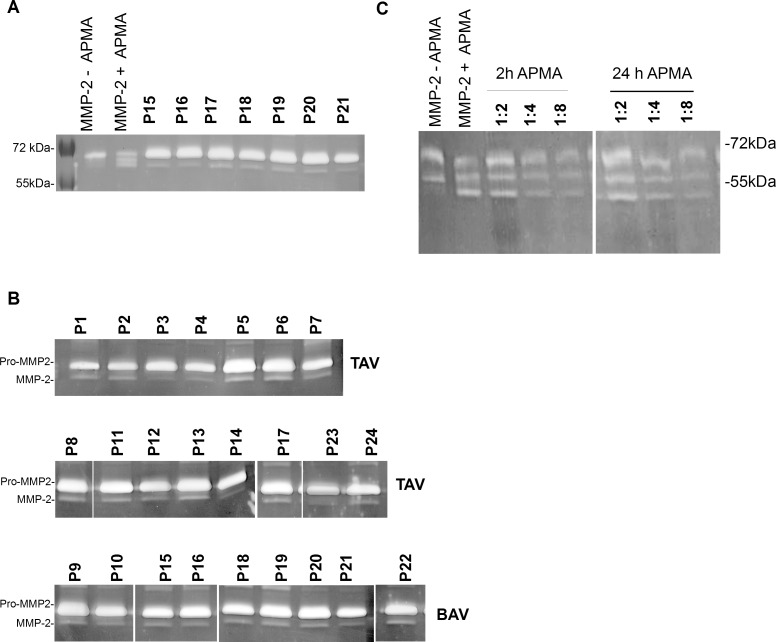
Representative zymograms showing gelatinolytic activity in protein extracts gained from aortic tissue of patients with ascending aortic/aortic root aneurysm. Protein extracts from patients with ascending aortic/aortic root aneurysms were analyzed for existence of different MMP-2 isoforms. Lane 1: Human full length MMP-2 as delivered. Lane 2: Human full length MMP-2 was activated with 2 mM APMA for 2 hours at 37°C prior to electrophoresis. Asterisk indicates 67 kDa intermediate MMP-2 isoform. **A.** 7 representative of 24 analyzed samples are shown. Signals corresponding to pro-MMP-2 were detected in every sample. Signals relatable to the active 65kDa MMP-2 were detected in all samples as well (see also S1 Fig). **B.** pro- and active MMP-2 in patients with bicuspid and tricuspid aortic valves. 15 of the analyzed samples were taken from patients with triicuspid aortic valves and 9 of the samples were from patients with bicuspid aortic valves. In both groups, gelatinolytic activities for either pro-MMP-2 and active MMP-2 were detectable. **C.** Activation of aortic tissue MMP-2. To test if tissue MMP-2 can be activated similar to the human full length MMP-2 used as a standard, protein extracts were incubated with 2mM APMA for either 2 or 24 hours. Different final dilutions of protein extracts were analyzed as indicated. Aortic tissue MMP-2 was split into three fragments similar to the human full length MMP-2. Extending APMA incubation time did not change the band pattern obtained after activation.

Zymography gels for Fig 1B and 1C were scanned immediately after staining. For Fig 1, contrast and brightness were enhanced. Gels for Fig 2B and 2C were scanned immediately after staining and for Fig 2, contrast was enhanced. Gels for Figs 3 and 4 were photographed on a light source to make signals visible and exposition was changed. Gels for Fig 4 were photographed on a light source, for Fig 4A, contrast was enhanced and for Fig 4 exposition was changed and brightness and contrast were adjusted to make weak signals visible. For the zymograms in Fig 5, contrast and brightness were changed. Gels for Fig 5C were photographed on a light source and exposition was changed. Gels used for semiquantitative determination of pro-MMP-2 were scanned with 600 dpi after staining and converted into 32-bit grayscale before measurements.

## Results

### Validation of gelatin zymography

Gelatin zymography was validated by running calibration curves with different amounts of human full length MMP-2 (0.0–2.5 ng). Human full length MMP-2 used as a standard was isolated from human rheumatoid synovial fibroblasts (Abcam technical service, personal communication). The signals observed in the zymograms decreased with declining MMP-2 amount. We also observed a distinct linear relationship between the measured pixel density and MMP-2 amount analyzed in the zymograms (Fig 1A and 1B). In addition, we analyzed dilution series of protein extracts gained from ascending aortic tissue from patients with ascending aortic aneurysms / aortic root aneurysms. In the respective zymograms, a decrease of the signals with increasing dilution factor was visible (Fig 1D). Four independent measurement showed, that the measured pixel densities decreased in a linear fashion between dilution factors of 2fold until 8fold. Also, the independent curves reveal, that gelatin zymography provides reproducible results. (Fig 1C and 1D).

Serum or tissue zymograms exhibited one (serum) or two (tissue) gelatinolytic activities. The serum gelatinolytic band and upper gelatinolytic band in the tissue samples corresponded to the main band at about 72 kDa observed in the human full length MMP-2 used as a standard. The upper band was confirmed to be MMP-2 by Westernblot analyses. (Fig 1E; S1 Fig; S2 Fig). In the tissue samples taken from aortic tissue from patients with ascending aortic aneurysms, we observed a second gelatinolytic activity at a molecular weight of approximately 65 kDa, where the active MMP-2 is known to run (Fig 1E; S2 Fig).

### Gelatin zymography detects only pro-MMP-2 in serum from patients with ascending aortic aneurysms or in healthy controls

We analyzed serum from 24 patients with ascending aortic aneurysms and 19 healthy controls by Enzyme-linked Immunosorbent Assay (ELISA) and gelatin zymography. ELISA results revealed, that serum MMP-2 levels were higher in control subjects (mean 240.3 +/- 46.4) than in patients with ascending aortic and aortic root aneurysms (mean = 190.3 +/- 48.3) with P = 0.0014 for both differences (Fig 2A).

The gelatin zymographic analysis of patient and control sera revealed only the existence of pro-MMP-2 in those samples. Pro-MMP-2 was identified via a human full length MMP-2 as a positive control (Fig 2B and 2C). Serum and human full length MMP-2 activated by incubation with APMA were also analyzed in each gel.

Activation of human full length MMP-2 resulted in pro-MMP-2 fragmentation into an intermediate 67 kDa form and the active 65 kDa form (Fig 2B and 2C, lane 1) Serum pro MMP-2 activated with APMA (as described for the human full length MMP-2) was split into the pro- and -intermediate forms only (Figs 2B and 2C lane 3 and 3B and 3D).

We analyzed increasing amounts of total protein of serum from patients and controls to exclude, that there were amounts of the 65 kDa active MMP-2 in the tested samples that were close to or below detection limit. Increasing total protein amounts until 80 μg did not lead to appearance of signals that could indicate presence of active MMP-2 in the tested samples from patients or healthy controls (Fig 3A and 3C).

Serum samples that had been incubated with APMA were also analyzed in increasing amounts to ensure that that no 65 kDa active MMP-2 became visible when loading higher amounts of serum onto the gels. Again, the separation of a total protein amount of 40 μg made no signals visible, that could be associated to the 65 kDa active MMP-2 (Fig 3B and 3D).

We also analyzed different incubation times of serum with APMA. Changing the serum`s incubation time with APMA did not alter the band pattern. A second band at an intermediate height of 67 kDa occurred at all of the different incubation periods chosen for serum MMP-2 activation in either patient serum (Fig 4A and 4C) or in that from healthy controls (Fig 4B). We detected no third band at 65 kDa as found in the human full length MMP-2 in any of the tested samples at increased incubation times with APMA (Fig 4).

### Aneurysmatic aortic tissue contains pro- and active MMP-2 irrespective of aortic valve morphology

We analyzed protein extracts taken from ascending aortic tissue from 24 patients with ascending aortic / aortic root aneurysms. Human full length MMP-2 and human full length MMP-2 activated with APMA were also analyzed in each gel (Fig 5A, lane 1 and 2). All tissue protein extracts taken from ascending aortic aneurysms contained considerable amounts of the 72kDa pro-MMP-2. We also noted considerable amounts of active 65 kDa MMP-2 in all tissue extracts from patients with ascending aortic aneurysms tested (Fig 5A and 5B). Pro- and active MMP-2 were measurable in protein extracts from patients with ascending aortic aneurysms with bicuspid aortic valves and in patients with ascending aortic aneurysms and tricuspid aortic valves (Fig 5B, S1 Fig). After incubating the aortic tissue extracts with APMA, we observed a different fragmentation pattern for tissue MMP-2 compared to serum MMP-2 (Fig 5C and Fig 4). Incubation of protein extracts taken from aortic tissue with APMA for 2 hours or for 24 hours resulted in fragmentation of the contained MMP-2 into three fragments similar to the human full length MMP-2 used as positive control (Fig 5C). Dilution of the protein extracts activated with APMA did not lead to disappearance of the signals for the active 65 kDa MMP-2 (Fig 5C).

### There is no significant correlation between MMP-2 isoforms in aortic tissue and aortic diameter

**Fig 6:**
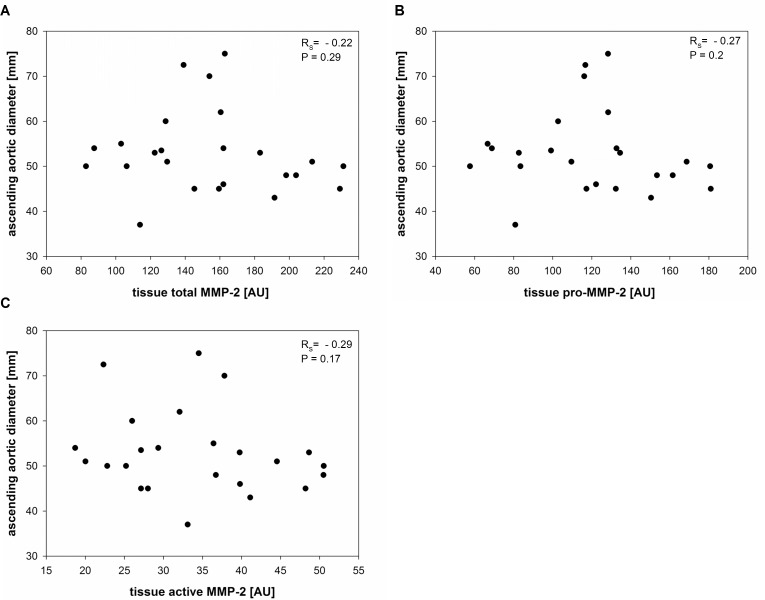
Correlation between tissue total-MMP-2, tissue pro-MMP-2 and tissue active- MMP-2 and ascending aortic diameter. Different MMP-2 isoforms were correlated to ascending aortic diameter to test, whether a certain MMP-2 isoform can represent changing mechanical properties in the expanding aortic wall. r_S_ = Spearman correlation coefficient; AU: Arbitrary units. **A.** Spearman correlation analysis of ascending aortic diameter and tissue total MMP-2 showed that there`s no significant relationship between both these parameters. Correlation coefficient reaches levels for a very weak negative correlation. (r_S_ = - -0.22; P = 0.29). **B.** Spearman correlation analysis of ascending aortic diameter and tissue pro-MMP-2 revealed no significant relationship between those two parameters. Correlation coefficient reaches levels for a very weak correlation. (r_S_ = -0.27; P = 0.2). **C.** Spearman correlation analysis of ascending aortic diameter and 65kDa active MMP-2 revealed no significant relationship between these two parameters. Correlation coefficient also reaches levels for a very weak correlation and is slightly higher as for tissue total MMP-2 and tissue pro-MMP-2 but without statistical significance. (r_S_ = -0.29; P = 0.17).

As the mechanical properties of the aortic wall were described to change with increasing aortic diameter [20], we decided to make correlation analyses for different MMP-2 isoforms to aortic diameter to find out whether there is a relationship between the changing mechanical properties of the aortic wall and a certain MMP-2 isoform. Values for pro-MMP-2, active MMP-2 and total MMP-2 in aortic tissue were analyzed by semiquantitative gelatin zymography. The values for tissue MMP-2 isoforms were normally distributed as calculated by Shapiro Wilk test. The values for aortic diameters were not normally distributed and therefore, spearman correlation coefficients were calculated to analyze relationships between values for tissue pro-MMP-2, tissue active MMP-2 and tissue total MMP-2 and aortic diameter. Total tissue MMP-2 was calculated by summarizing the values for tissue pro-MMP-2 and tissue active MMP-2. Our analyses revealed no significant correlation between total tissue MMP-2 and aortic diameter or between tissue pro-MMP-2 and aortic diameter (r_S_ = -0.22; P = 0.29 and r_S_ = 0.27; P = 0.2). The data distribution of pro-MMP-2 and total MMP-2 looks very similar. The correlation coefficients show, that there is a very weak negative relationship between tissue pro-MMP-2 and tissue total MMP-2 and aortic diameter without statistical significance. (Fig 6A and 6B). Furthermore, we identified no significant correlation between active MMP-2 and aortic diameter (r_S_ = 0.29; P = 0.17; Fig 6C). The Spearman correlation coefficient for active MMP-2 attained levels for indicating a very weak negative correlation either. But there was no statistical significance.

### There`s no correlation between tissue and serum MMP-2

**Fig 7:**
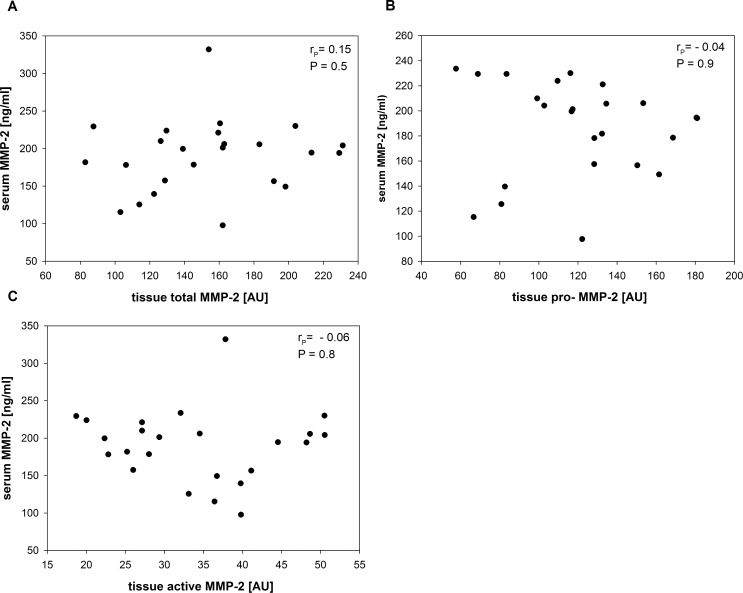
Correlation between serum MMP-2 and different tissue MMP-2 isoforms Pearson`s correlation analyses between serum MMP-2 and different tissue MMP-2 isoforms were performed to test for any a measurable linear connection between tissue and serum MMP-2. r_P_ = Pearson correlation coefficient; AU: arbitrary units. **A.** Pearson correlation analysis revealed no significant linear relationship between serum MMP-2 and total tissue MMP-2 (r_P_ = - 0.15; P = 0.5). Correlation coefficient is greater than for tissue pro-MMP-2 and tissue active MMP-2 but without a statistical significance. **B.** Pearson correlation demonstrated no significant linear relationship between serum MMP-2 and tissue pro-MMP-2 (r_P_ = - 0.04; P = 0.9). Correlation coefficient is close to zero. **C.** Pearson correlation analysis showed no significant linear relationship between serum MMP-2 and tissue active MMP-2 (r_P_ = - 0.06; P = 0.8). Correlation coefficient is almost zero.

Serum MMP-2 levels could be associated to ascending aortic aneurysms in a previous study [24] and we found, that serum MMP-2 levels for patients with ascending aortic aneurysms significantly lower than serum levels in healthy controls (190.3 +/- 48.3 ng/ml vs 240.3 +/- 46.4 ng/ml). To connect serum MMP-2 to tissue MMP-2 and to analyze whether serum MMP-2 can be related to a tissue MMP-2 isoform, we conducted correlation analyses for tissue pro-MMP-2, tissue total MMP-2 and tissue active MMP-2. Serum MMP-2 levels and levels of tissue MMP-2 isoforms were normally distributed (Shapiro Wilk test). Therefore, we analyzed serum MMP-2 and tissue MMP-2 isoforms by pearson`s correlation analysis for a linear relationship between serum and tissue MMP-2. To detect any indication, that tissue–MMP-2 and serum—MMP-2 might be interrelated, we also correlated tissue MMP-2 isoforms to serum MMP-2. There was no correlation between serum and total tissue MMP-2 (r_P_ = 0.15; P = 0.5) (Fig 7A). The correlation coefficient indicated a very low correlation but without statistical significance. Since pro-MMP-2 was the only MMP-2 isoform detectable in serum, we also measured pro-MMP-2 alone and correlated tissue pro-MMP-2 to serum MMP-2 finding no correlation (r_P_ = - 0.04; P = 0.9) (Fig 7B). Correlation analysis of serum MMP-2 and tissue active MMP-2 revealed no significant linear relationship between these two parameters either (r_P_ = - 0.06; P = 0.8: Fig 7C). The correlation coefficients for pro-MMP-2 and active MMP-2 and aortic diameter were close to zero indicating not even a weak linear relationship.

## Discussion

Aneurysm progression is a very dynamic and complex anomaly and numerous factors are known to be involved in this process [25] [26] [27]. These are genetic factors linked to TGF β aortic valve configuration and altered haemodynamics. [28] [29] [30] [27]. In addition, processes known to be associated to aneurysm formation like elastin degradation also occur under physiological conditions [31].

Our finding, that patient sera contained slightly lower MMP-2 amounts than control sera is in line with that complexity Our results differ from previous findings, where MMP-2 plasma levels were elevated in association with ascending aortic aneurysms [24]. In another study, plasma levels of MMP-2 were found to be lower in patients with ascending aortic aneurysms. [32]. These discrepancies may be due to different group sizes. But they also reflect the heterogeneity of the data available on MMP-2 and highlight the need to analyze a potential biomarker like MMP-2 in greater detail.

MMP-2 was associated to ascending aortic aneurysms in both aortic tissue and serum [32] [24] [33].[34] Until now, no one had tried to investigate serum and tissue isoforms of MMP-2 simultaenously to test whether isoforms in these sample types are interconnected.

Pro-MMP-2 was the only MMP-2 species we could identify in patient or control serum via gelatin zymography. The presence of exclusively pro-MMP-2 in control serum is in is well in line with findings in the literature [35].

Our controls revealed no evidence, that active MMP-2 in patient serum is present but below the detection limit. Of course, it is possible that there may have been very faint signals of active MMP-2 in patient serum, that were not detectable under our experimental conditions. However, we detected no correlation between tissue and serum MMP-2, irrespective of the isoform tested.

We could not generate the active 65 kDa MMP-2 form by APMA activation of pro-MMP-2 in patient or control serum. In contrast, human full length MMP-2 isolated from rheumatoid synovial fibroblasts (Abcam technical service, personal communication) as well as aortic tissue MMP-2 was clearly split into three forms containing the initial pro-MMP-2 (72 kDa), the intermediate form (67 kDa) and MMP-2 (65 kDa). Controls yielded no hint that 65 kDa MMP-2 was present in serum after APMA activation but below the detection limit or that serum MMP-2 activation takes longer than 2 hours. These differences can point to MMP-2/TIMP interactions in serum, as these were shown to influence the formation or stability of different MMP-2 isoforms [36]. Therefore further investigation is required to discover whether there are different MMP-2/TIMP interactions in the serum of patients with ascending aortic aneurysms versus healthy controls.

The fact, that pro-MMP-2 was present in all our control sera and in serum from healthy controls in other studies [24] [32] also illustrates, that serum MMP-2 has origins apart from aortic aneurysms.

Animal models analyzing aneurysm progression [4] [37] reported that MMP-2 plays an important role in pathological ECM remodeling in aneurysmatic aortic tissue. We subjected aortic tissue MMP-2 to semiquantitative gelatin zymographic analysis to discover any indication that different MMP-2 isoforms can be associated to changing mechanical properties in the aortic wall and whether there is a linear relationship between tissue and serum MMP-2 isoforms. There is evidence of a linear relationship between pixel density measured on zymograms and MMP-2 enzyme amount [38]. We reproduced this linear relationship for our experimental system showing, that this approach is suitable for measuring MMP-2 isoforms. Analysis of several dilution series of protein extracts also confirmed reproducibility of the assay.

We observed, that pro-MMP-2 (72 kDa) and active MMP-2 (65 kDa) were detectable in all of our tissue samples taken from patients with ascending aortic aneurysms–irrespective of aortic valve configuration or other clinical parameters. The observation, that active MMP-2 cannot be measured in patient serum but is present in aortic tissue indicates, that proteins present in aneurysmatic tissue are not necessarily released into systemic circulation in detectable amounts or in a stable form. We detected pro and active MMP-2 in protein extracts from patients with bicuspid and tricuspid aortic valves but the levels of different MMP-2 isoforms have to be analyzed in a larger patient cohort as a t-test applied to compare levels of MMP-2 isoforms between patients with bicuspid and tricuspid aortic valves had a low power and the results were not interpretable.

As mechanical stress in the aortic wall increases with aortic diameter and pro-MMP-2 was shown to be released from cultivated VSMC`s in response to mechanical stretch, (regarded as one form of mechanical stress) [20] [17] and high wall shear stress was shown to stimulate pro- and active MMP-2 [19], we aimed to discover whether aortic tissue -MMP-2 isoforms can be related to aortic diameter.

We could, however, identify no significant correlation between aortic diameter and pro-MMP-2, active MMP-2 or total MMP-2, showing, that diameter-related mechanical stress cannot directly be linked to a specific MMP-2 isoform in aortic tissue.

We detected no linear correlation between serum MMP-2 and aortic tissue MMP-2, nor between total tissue MMP-2 / serum MMP-2, nor between tissue pro-MMP-2 /serum MMP-2, nor between tissue active MMP-2 and serum MMP-2. These findings indicate, that the lack of a correlation between serum and tissue MMP-2 is not due to measuring of different MMP-2 isoforms in both sample types.

We detected no sign that serum and tissue MMP-2 are interconnected or that an increased aortic diameter with increased wall stress is linked to a greater pro-MMP-2 or a greater active-MMP-2 availability in the analyzed tissue samples. Taken together, this indicates, that MMP-2 alone is an unsuitable marker for a common measurement of high wall stress in ascending aortic aneurysms. Both VSMC`s and endothelial cells (EC`s) have been shown to release MMP-2 [39] [17] and both cell types detect different types of mechanical forces. Whereas EC`s are mainly exposed to shear stress, VSMC`s tend to be subjected to stretch. These two factors are indistinguishable in aortic tissue which is why the effect of mechanical forces on matrix degrading enzymes like MMP-2 should undergo further analyzis in cell cultures. Especially the fact, that pro-MMP-2 / aortic diameter and active MMP-2 /aortic diameter showed weak negative correlation coefficients without statistical significance indicates, that the relationship between mechanical forces and a possible release and activation of matrix degrading enzymes like MMP-2 needs a more detailed analysis under controlled experimental conditions.

We also need to elucidate whether pro- or active MMP-2 levels in the aortic wall or in serum can be related to individual blood flow patterns and aortic geometry, as these factors may trigger individual forms of wall stress that cannot be assessed by measuring aortic diameter alone. This study is limited by its rather small patient groups, subgroups of patients with ascending aortic aneurysms, especially patients with bicuspid and tricuspid aortic valves should be investigated in a larger study to evaluate the potential of MMP-2 isoforms as indicators of high wall stress or to definitely rule out the suitability of MMP-2 as such a marker.

## Supporting Information

S1 Fig Zymograms of all protein extracts taken from ascending aortic tissue tested in this study. 24 protein extracts gained from ascending aortic tissue from patients with ascending aortic/aortic root aneurysms were analyzed by gelatin zymography. Human full length MMP-2 (ab168864, Abcam) was analyzed as a control. In Addition, human full length MMP-2 was activated by incubation with APMA for 2 hours at 37°C. 1: Human full length MMP-2 as delivered. Human full length MMP-2 showed signals at about 70 kDa where pro-MMP-2 would be expected. A minor band that corresponded to intermediate MMP-2 was also detected. 2: Human full length MMP-2 incubated with APMA for 2 hours at 37°C. Activation of human full length MMP-2 led to fragmentation of pro-MMP-2 into an additional intermediate form and active MMP-2 at about 65 kDa. P1—P24. protein extracts from patient 1–24; asterisk indicates samples from patients with bicuspid aortic valves.
(PPTX)

S2 Fig Westernblot confirmed existence of pro-MMP-2 in protein extracts gained from ascending aortic tissue. Westernblots were performed with protein extracts gained from the same aortic tissue samples as in the zymograms. The blots show a distinct signal at about 70 kDa for the human full length MMP-2 which was also present in each protein extract analyzed. 1: MMP-2 standard (human full length MMP-2). P1—P24: protein extracts from aortic tissue from patient 1–24.
(PPTX)

S3 Fig Zymograms of all patient and control sera analyzed in this study. All serum samples tested in this study showed a signal at the same height as the main signal in the human full length MMP-2. A weak signal for the intermediate MMP-2 isoform was also seen in the human full length MMP-2 as delivered. 1: Human full length MMP-2 as delivered. Human full length MMP-2 showed a strong signal at about 70 kDa where pro-MMP-2 is expected. A minor band at an intermediate height of approximately 67 kDa was also present in the human full length. MMP-2 without additional activation. 2: Human full length MMP-2 incubated with APMA for 2 hours at 37°C. The incubation led to fragmentation of the human full length MMP-2 into three signals at about 70kDa, 67kDa and 65kDa. 3: Serum incubated with 2 mM APMA for 2 hours at 37°C. APMA incubation of serum led to fragmentation of the contained MMP-2 into pro- and intermediate MMP-2 only. **A:** Zymograms showing gelatinolytic activities in serum from patients with ascending aortic aneurysms. P1—P24. serum from patient 1–24. Serum samples from the same patients as analyzed for the tissue showed a signal corresponding to pro-MMP-2 only. **B:** Zymograms showing gelatinolytic activities in serum from healthy controls. C1-C19: Serum from control 1–19.
(PPTX)

S1 Table Relationship between MMP-2 amount loaded on zymograms and pixel density. Different amounts of human full length MMP-2 were loaded onto the zymogram gels and analyzed as described. The values represent total pixel densities measured in different zymograms. Each amount was analyzed twice in the same gel.
(PPTX)

S2 Table Relationship between dilution factor and pixel density. Protein extracts from ascending aortic tissue were diluted as indicated. Average pixel densitiy represents the mean of one sample run twice at in the same gel. StDev: Standard Deviation.
(PPTX)

S3 Table Serum MMP-2 levels in patients and controls. Serum MMP-2 was measured by Enzyme-linked Immunosorbent Assay.
(PPTX)

S4 Table Pixel densities measured in protein extracts from aortic tissue from patients with ascending aortic aneurysms. A: Experiment 1; B. Experiment 2; C: Experiment 3.
(PPTX)

S5 Table MMP-2 pixel densities used for calculation of average pixel density of pro-MMP-2, active MMP-2 and total MMP-2. Each standard value represents 0.33 ng human full length MMP-2.
(PPTX)

S6 Table MMP-2 values and ascending aortic diameter used for correlation analyses. MMP-2 values from zymograms were calculated as described in Material and methods.
(PPTX)

## References

[1] Rylski B, Blanke P, Beyersdorf F, Desai ND, Milewski RK, Siepe M, et al How does the ascending aorta geometry change when it dissects? J Am Coll Cardiol. 2014;63: 1311–1319. 10.1016/j.jacc.2013.12.028 24509277

[2] Barbour JR, Spinale FG, Ikonomidis JS. Proteinase systems and thoracic aortic aneurysm progression. J Surg Res. 2007;139: 292–307. 10.1016/j.jss.2006.09.020 17292415

[3] Visse R, Nagase H. Matrix Metalloproteinases and Tissue Inhibitors of Metalloproteinases Structure, Function, and Biochemistry. Circ Res. 2003;92: 827–839. 10.1161/01.RES.0000070112.80711.3D 12730128

[4] Eckhouse SR, Logdon CB, Oelsen JM, Patel RK, Rice AD, Stroud RE, et al Reproducible Porcine Model of Thoracic Aortic Aneurysm. Circulation. 2013;128: S186–S193. 10.1161/CIRCULATIONAHA.112.000363 24030405 PMC3840947

[5] Ikonomidis JS, Ruddy JM, Benton SM Jr, Arroyo J, Brinsa TA, Stroud RE, et al Aortic Dilatation With Bicuspid Aortic Valves: Cusp Fusion Correlates to Matrix Metalloproteinases and Inhibitors. Ann Thorac Surg. 2012;93: 457–463. 10.1016/j.athoracsur.2011.09.057 22206960 PMC3265643

[6] Satta J, Juvonen T, Haukipuro K, Juvonen M, Kairaluoma MI. Increased turnover of collagen in abdominal aortic aneurysms, demonstrated by measuring the concentration of the aminoterminal propeptide of type III procollagen in peripheral and aortal blood samples. J Vasc Surg. 1995;22: 155–160. 10.1016/S0741-5214(95)70110-9 7637115

[7] Anidjar S, Salzmann JL, Gentric D, Lagneau P, Camilleri JP, Michel JB. Elastase-induced experimental aneurysms in rats. Circulation. 1990;82: 973–981. 10.1161/01.CIR.82.3.973 2144219

[8] Longo GM, Xiong W, Greiner TC, Zhao Y, Fiotti N, Baxter BT. Matrix metalloproteinases 2 and 9 work in concert to produce aortic aneurysms. J Clin Invest. 2002;110: 625–632. 10.1172/JCI15334 12208863 PMC151106

[9] Vine N, Powell JT. Metalloproteinases in degenerative aortic disease. Clin Sci. 1991;81: 233–239. 10.1042/cs0810233 1653668

[10] Sudhakar YA, Verma RK, Pawar SC. Type IV collagen α1-chain noncollagenous domain blocks MMP-2 activation both in-vitro and in-vivo. Sci Rep. 2014;4: 4136 10.1038/srep04136 24670518 PMC3966261

[11] English JL, Kassiri Z, Koskivirta I, Atkinson SJ, Grappa MD, Soloway PD, et al Individual Timp Deficiencies Differentially Impact Pro-MMP-2 Activation. J Biol Chem. 2006;281: 10337–10346. 10.1074/jbc.M512009200 16469749

[12] Arpino V, Brock M, Gill SE. The role of TIMPs in regulation of extracellular matrix proteolysis. Matrix Biol. 2015;44–46: 247–254. 10.1016/j.matbio.2015.03.005 25805621

[13] Strongin AY, Collier I, Bannikov G, Marmer BL, Grant GA, Goldberg GI. Mechanism of cell surface activation of 72-kDa type IV collagenase. Isolation of the activated form of the membrane metalloprotease. J Biol Chem. 1995;270: 5331–5338. 7890645 10.1074/jbc.270.10.5331

[14] Sternlicht MD, Werb Z. How Matrix Metalloproteinases Regulate Cell Behavior. Annu Rev Cell Dev Biol. 2001;17: 463–516. 10.1146/annurev.cellbio.17.1.463 11687497 PMC2792593

[15] Kimura K, Cheng XW, Nakamura K, Inoue A, Hu L, Song H, et al Matrix metalloproteinase-2 regulates the expression of tissue inhibitor of matrix metalloproteinase-2. Clin Exp Pharmacol Physiol. 2010;37: 1096–1101. 10.1111/j.1440-1681.2010.05441.x 20738326

[16] Shen M, Lee J, Basu R, Sakamuri SSVP, Wang X, Fan D et al Divergent Role of Matrix Metalloproteinase 2 in Pathogenesis of Thoracic Aortic Aneurysm. Arterioscler Thromb Vasc Biol. 2015; ATVBAHA.114.305115. 10.1161/ATVBAHA.114.305115 25657308

[17] Grote K, Flach I, Luchtefeld M, Akin E, Holland SM, Drexler H, et al Mechanical Stretch Enhances mRNA Expression and Proenzyme Release of Matrix Metalloproteinase-2 (MMP-2) via NAD(P)H Oxidase–Derived Reactive Oxygen Species. Circ Res. 2003;92: e80–e86. 10.1161/01.RES.0000077044.60138.7C 12750313

[18] Guzzardi DG, Barker AJ, van Ooij P, Malaisrie SC, Puthumana JJ, Belke DD, et al Valve-Related Hemodynamics Mediate Human Bicuspid Aortopathy: Insights From Wall Shear Stress Mapping. J Am Coll Cardiol. 2015;66: 892–900. 10.1016/j.jacc.2015.06.1310 26293758 PMC4545965

[19] Tronc F, Mallat Z, Lehoux S, Wassef M, Esposito B, Tedgui A. Role of matrix metalloproteinases in blood flow-induced arterial enlargement: interaction with NO. Arterioscler Thromb Vasc Biol. 2000;20: E120–126. 11116076 10.1161/01.atv.20.12.e120

[20] Koullias G, Modak R, Tranquilli M, Korkolis DP, Barash P, Elefteriades JA. Mechanical deterioration underlies malignant behavior of aneurysmal human ascending aorta. J Thorac Cardiovasc Surg. 2005;130: 677–683. 10.1016/j.jtcvs.2005.02.052 16153912

[21] Kari FA, Kocher N, Beyersdorf F, Tscheuschler A, Meffert P, Rylski B, et al Four-dimensional magnetic resonance imaging-derived ascending aortic flow eccentricity and flow compression are linked to aneurysm morphology†. Interact Cardiovasc Thorac Surg. 2015;20: 582–587; discussion 587–588. 10.1093/icvts/ivu446 25636325 PMC4626745

[22] Toth M, Sohail A, Fridman R. Assessment of gelatinases (MMP-2 and MMP-9) by gelatin zymography. Methods Mol Biol Clifton NJ. 2012;878: 121–135. 10.1007/978-1-61779-854-2_8 22674130

[23] Towbin H, Staehelin T, Gordon J. Electrophoretic transfer of proteins from polyacrylamide gels to nitrocellulose sheets: procedure and some applications. Proc Natl Acad Sci U S A. 1979;76: 4350–4354. 388439 10.1073/pnas.76.9.4350 PMC411572

[24] Huusko T, Salonurmi T, Taskinen P, Liinamaa J, Juvonen T, Pääkkö P, et al Elevated messenger RNA expression and plasma protein levels of osteopontin and matrix metalloproteinase types 2 and 9 in patients with ascending aortic aneurysms. J Thorac Cardiovasc Surg. 2013;145: 1117–1123. 10.1016/j.jtcvs.2012.04.008 22571802

[25] Ruddy JM, Jones JA, Ikonomidis JS. Pathophysiology of thoracic aortic aneurysm (TAA): is it not one uniform aorta? Role of embryologic origin. Prog Cardiovasc Dis. 2013;56: 68–73. 10.1016/j.pcad.2013.04.002 23993239 PMC3759819

[26] El-Hamamsy I, Yacoub MH. Cellular and molecular mechanisms of thoracic aortic aneurysms. Nat Rev Cardiol. 2009;6: 771–786. 10.1038/nrcardio.2009.191 19884902

[27] Humphrey JD, Schwartz MA, Tellides G, Milewicz DM. Role of Mechanotransduction in Vascular Biology Focus on Thoracic Aortic Aneurysms and Dissections. Circ Res. 2015;116: 1448–1461. 10.1161/CIRCRESAHA.114.304936 25858068 PMC4420625

[28] Saratzis A, Bown MJ. The genetic basis for aortic aneurysmal disease. Heart Br Card Soc. 2014;100: 916–922. 10.1136/heartjnl-2013-305130 24842835

[29] Verma S, Siu SC. Aortic Dilatation in Patients with Bicuspid Aortic Valve. N Engl J Med. 2014;370: 1920–1929. 10.1056/NEJMra1207059 24827036

[30] Atkins SK, Cao K, Rajamannan NM, Sucosky P. Bicuspid aortic valve hemodynamics induces abnormal medial remodeling in the convexity of porcine ascending aortas. Biomech Model Mechanobiol. 2014; 10.1007/s10237-014-0567-7 24599392

[31] Schlatmann TJM, Becker AE. Histologic changes in the normal aging aorta: Implications for dissecting aortic aneurysm. Am J Cardiol. 1977;39: 13–20. 10.1016/S0002-9149(77)80004-0 831420

[32] Ikonomidis JS, Ivey CR, Wheeler JB, Akerman AW, Rice A, Patel RK, et al Plasma biomarkers for distinguishing etiological subtypes of thoracic aortic aneurysm disease. J Thorac Cardiovasc Surg. 2013;145: 1326–1333. 10.1016/j.jtcvs.2012.12.027 23312977 PMC4057430

[33] Jones JA, Stroud RE, O’Quinn EC, Black LE, Barth JL, Elefteriades JA, et al Selective microRNA Suppression in Human Thoracic Aneurysms: Relationship of miR-29a to Aortic Size and Proteolytic Induction. Circ Cardiovasc Genet. 2011;4: 605–613. 10.1161/CIRCGENETICS.111.960419 22010139 PMC3246193

[34] LeMaire SA, Wang X, Wilks JA, Carter SA, Wen S, Won T, et al Matrix metalloproteinases in ascending aortic aneurysms: bicuspid versus trileaflet aortic valves. J Surg Res. 2005;123: 40–48. 10.1016/j.jss.2004.06.007 15652949

[35] Rocca GL, Pucci-Minafra I, Marrazzo A, Taormina P, Minafra S. Zymographic detection and clinical correlations of MMP-2 and MMP-9 in breast cancer sera. Br J Cancer. 2004;90: 1414–1421. 10.1038/sj.bjc.6601725 15054465 PMC2409673

[36] English JL, Kassiri Z, Koskivirta I, Atkinson SJ, Grappa MD, Soloway PD, et al Individual Timp Deficiencies Differentially Impact Pro-MMP-2 Activation. J Biol Chem. 2006;281: 10337–10346. 10.1074/jbc.M512009200 16469749

[37] Ikonomidis JS, Gibson WC, Gardner J, Sweterlitsch S, Thompson RP, Mukherjee R, et al A murine model of thoracic aortic aneurysms. J Surg Res. 2003;115: 157–163. 10.1016/S0022-4804(03)00193-8 14572787

[38] Peake NJ, Foster HE, Khawaja K, Cawston TE, Rowan AD. Assessment of the clinical significance of gelatinase activity in patients with juvenile idiopathic arthritis using quantitative protein substrate zymography. Ann Rheum Dis. 2006;65: 501–507. 10.1136/ard.2005.039032 16150790 PMC1798108

[39] Kopaliani I, Martin M, Zatschler B, Bortlik K, Müller B, Deussen A. Cell-specific and endothelium-dependent regulations of matrix metalloproteinase-2 in rat aorta. Basic Res Cardiol. 2014;109: 419 10.1007/s00395-014-0419-8 24907869

