# Supplementary material for: MMP-2 Isoforms in Aortic Tissue and Serum of Patients with Ascending Aortic Aneurysms and Aortic Root Aneurysms
Source: PLoS One. 2016 Nov 1;11(11):e0164308. doi: 10.1371/journal.pone.0164308 (PMC5089694; doi:10.1371/journal.pone.0164308)
Supplement: S1 Fig — 24 protein extracts gained from ascending aortic tissue from patients with ascending aortic/aortic root aneurysms were analyzed by gelatin zymography. Human full length MMP-2 (ab168864, Abcam) was analyzed as a control. In Addition, human full length MMP-2 was activated by incubation with APMA for 2 hours at 37°C. 1: Human full length MMP-2 as delivered. Human full length MMP-2 showed signals at about 70 kDa where pro-MMP-2 would be expected. A minor band that corresponded to intermediate MMP-2 was also detected. 2: Human full length MMP-2 incubated with APMA for 2 hours at 37°C. Activation of human full length MMP-2 led to fragmentation of pro-MMP-2 into an additional intermediate form and active MMP-2 at about 65 kDa. P1—P24. protein extracts from patient 1–24; asterisk indicates samples from patients with bicuspid aortic valves. (PPTX) [file pone.0164308.s001.pptx]

## Slide 1
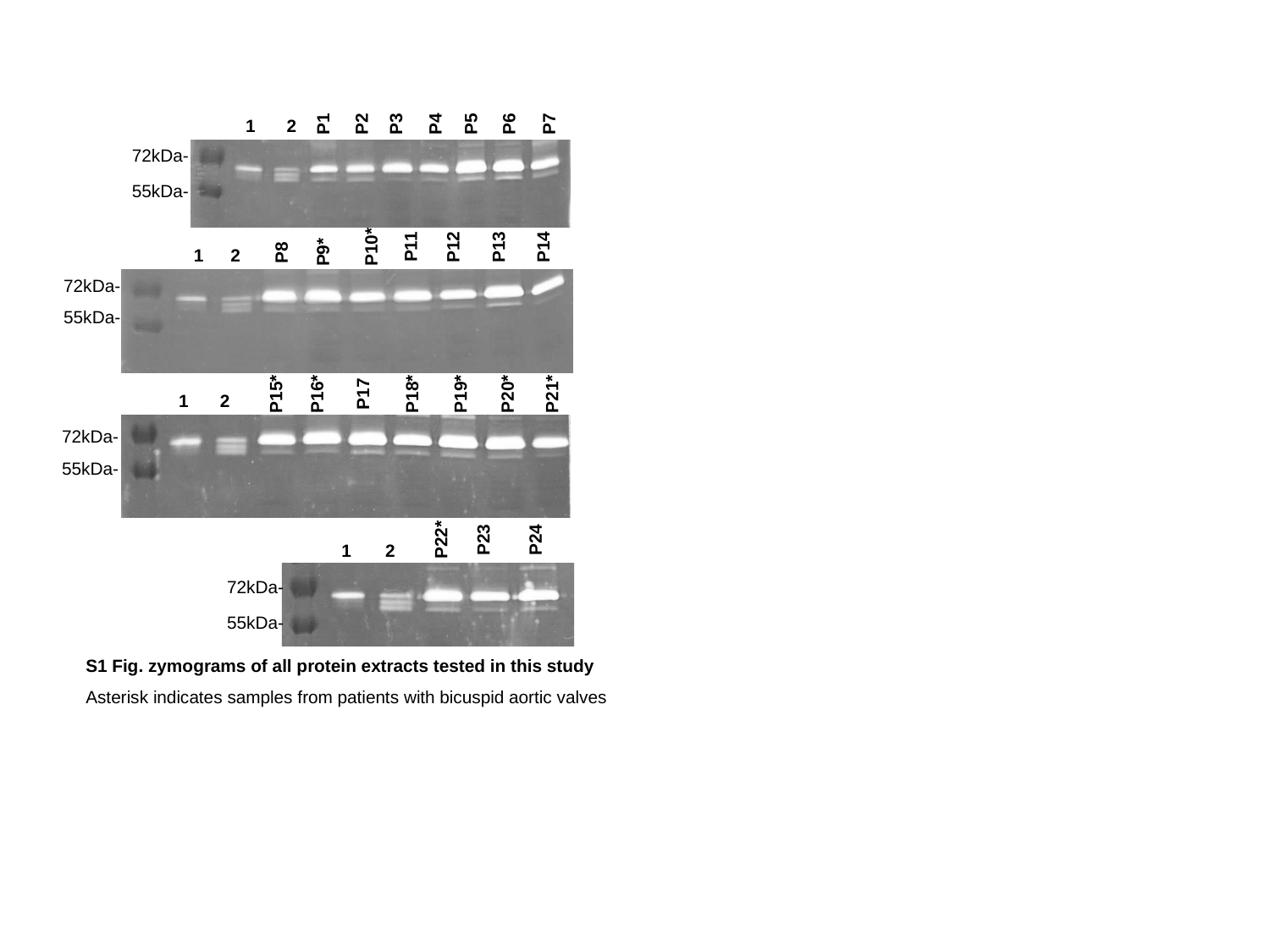

P1
P2
P3
P4
P5
P6
P7
1
2
72kDa-
55kDa-
P11
P12
P13
P14
P10*
P8
P9*
1
2
72kDa-
55kDa-
P17
P15*
P16*
P18*
P19*
P20*
P21*
1
2
72kDa-
55kDa-
P23
P24
P22*
1
2
72kDa-
55kDa-
S1 Fig. zymograms of all protein extracts tested in this study
Asterisk indicates samples from patients with bicuspid aortic valves
