# Supplementary material for: MMP-2 Isoforms in Aortic Tissue and Serum of Patients with Ascending Aortic Aneurysms and Aortic Root Aneurysms
Source: PLoS One. 2016 Nov 1;11(11):e0164308. doi: 10.1371/journal.pone.0164308 (PMC5089694; doi:10.1371/journal.pone.0164308)
Supplement: S2 Fig — Westernblots were performed with protein extracts gained from the same aortic tissue samples as in the zymograms. The blots show a distinct signal at about 70 kDa for the human full length MMP-2 which was also present in each protein extract analyzed. 1: MMP-2 standard (human full length MMP-2). P1—P24: protein extracts from aortic tissue from patient 1–24. (PPTX) [file pone.0164308.s002.pptx]

## Slide 1
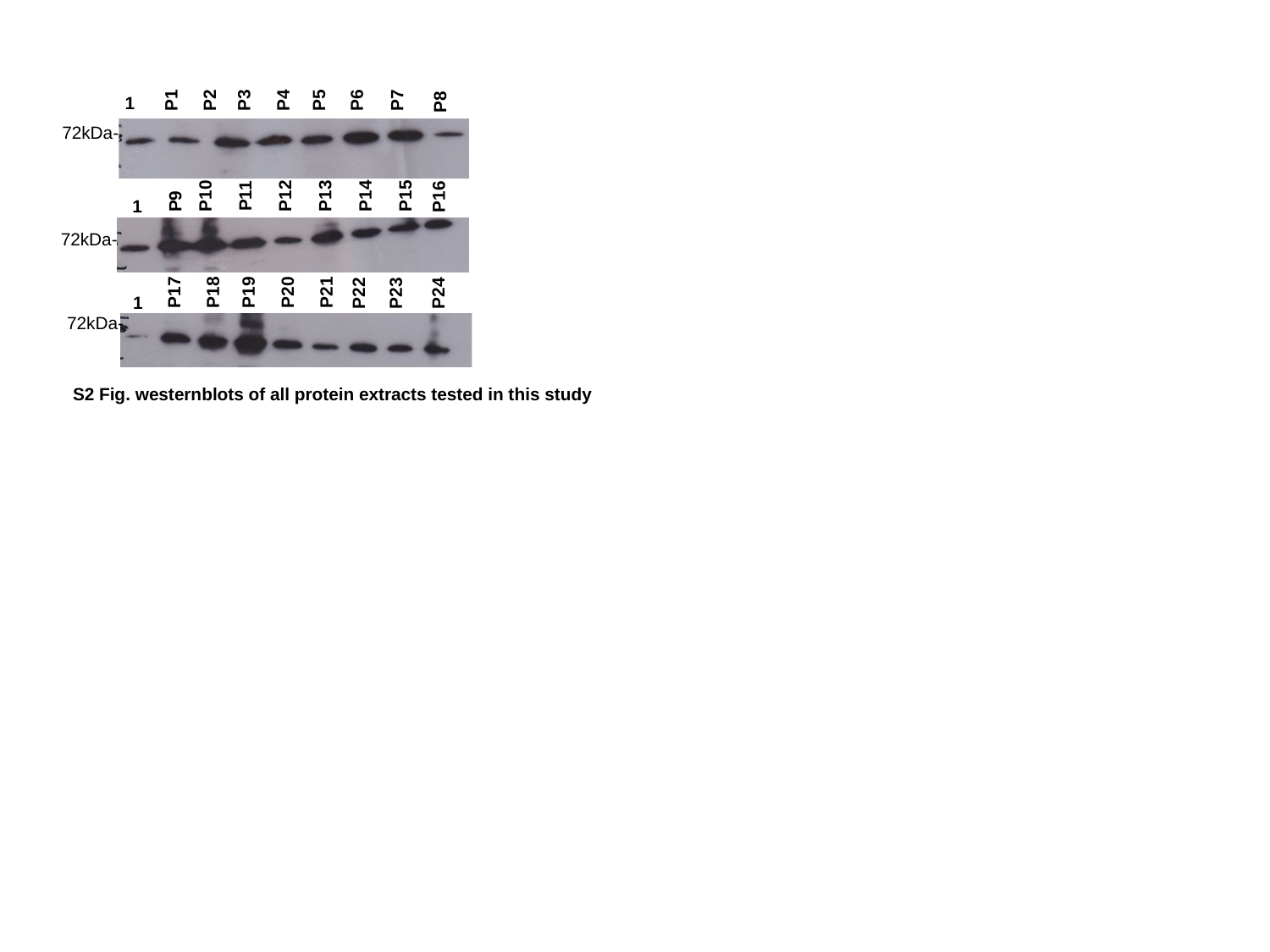

P1
P2
P3
P4
P5
P6
P7
P8
1
72kDa-
P10
P11
P12
P13
P14
P15
P16
P9
1
72kDa-
P17
P18
P19
P20
P21
P22
P23
P24
1
72kDa-
S2 Fig. westernblots of all protein extracts tested in this study
