# Supplementary material for: MMP-2 Isoforms in Aortic Tissue and Serum of Patients with Ascending Aortic Aneurysms and Aortic Root Aneurysms
Source: PLoS One. 2016 Nov 1;11(11):e0164308. doi: 10.1371/journal.pone.0164308 (PMC5089694; doi:10.1371/journal.pone.0164308)
Supplement: S3 Fig — All serum samples tested in this study showed a signal at the same height as the main signal in the human full length MMP-2. A weak signal for the intermediate MMP-2 isoform was also seen in the human full length MMP-2 as delivered. 1: Human full length MMP-2 as delivered. Human full length MMP-2 showed a strong signal at about 70 kDa where pro-MMP-2 is expected. A minor band at an intermediate height of approximately 67 kDa was also present in the human full length. MMP-2 without additional activation. 2: Human full length MMP-2 incubated with APMA for 2 hours at 37°C. The incubation led to fragmentation of the human full length MMP-2 into three signals at about 70kDa, 67kDa and 65kDa. 3: Serum incubated with 2 mM APMA for 2 hours at 37°C. APMA incubation of serum led to fragmentation of the contained MMP-2 into pro- and intermediate MMP-2 only. A: Zymograms showing gelatinolytic activities in serum from patients with ascending aortic aneurysms. P1—P24. serum from patient 1–24. Serum samples from the same patients as analyzed for the tissue showed a signal corresponding to pro-MMP-2 only. B: Zymograms showing gelatinolytic activities in serum from healthy controls. C1-C19: Serum from control 1–19. (PPTX) [file pone.0164308.s003.pptx]

## Slide 1
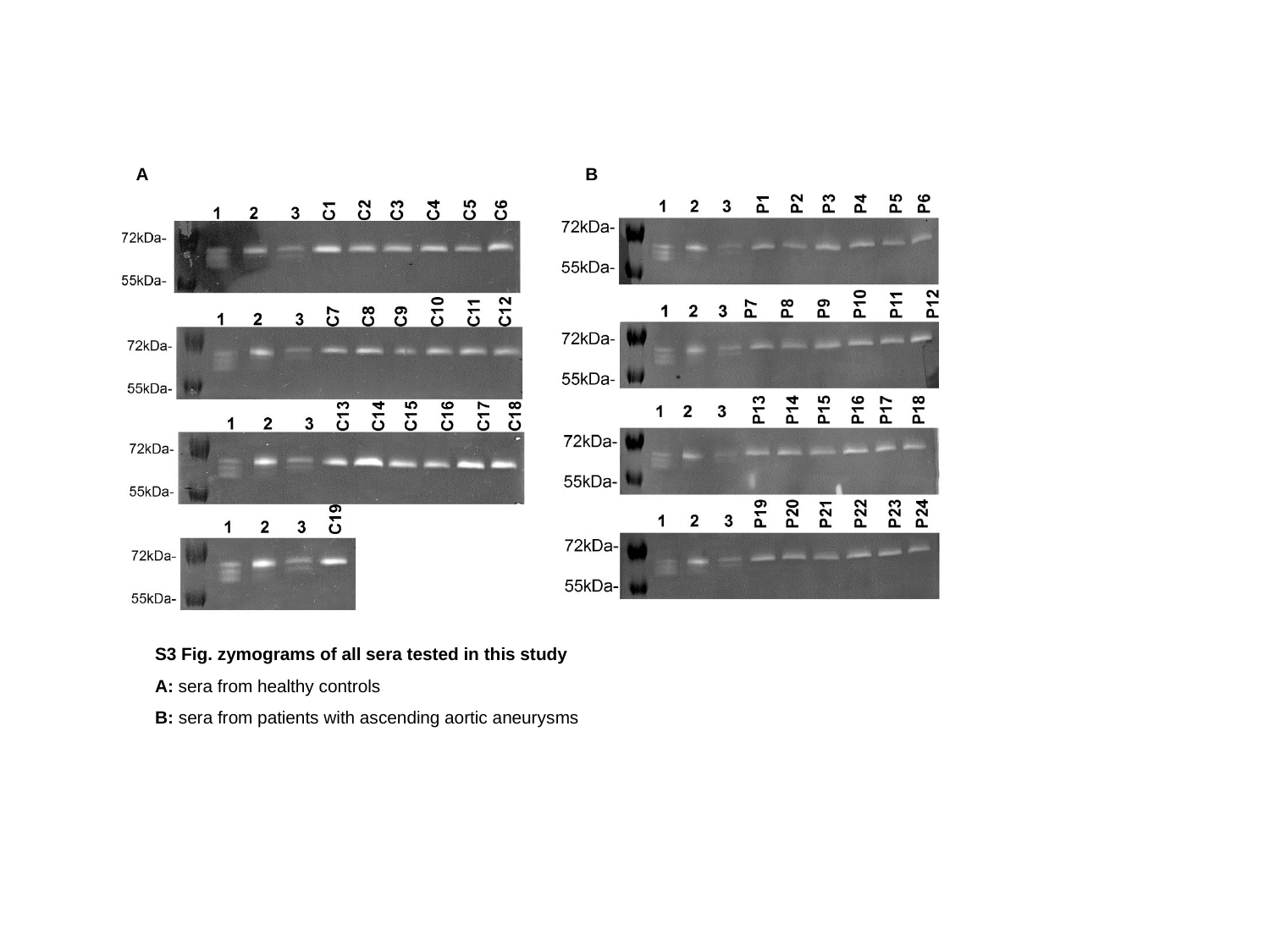

B
A
S3 Fig. zymograms of all sera tested in this study
A: sera from healthy controls
B: sera from patients with ascending aortic aneurysms
