# Supplementary material for: MMP-2 Isoforms in Aortic Tissue and Serum of Patients with Ascending Aortic Aneurysms and Aortic Root Aneurysms
Source: PLoS One. 2016 Nov 1;11(11):e0164308. doi: 10.1371/journal.pone.0164308 (PMC5089694; doi:10.1371/journal.pone.0164308)
Supplement: S1 Table — Different amounts of human full length MMP-2 were loaded onto the zymogram gels and analyzed as described. The values represent total pixel densities measured in different zymograms. Each amount was analyzed twice in the same gel. (PPTX) [file pone.0164308.s004.pptx]

## Slide 1
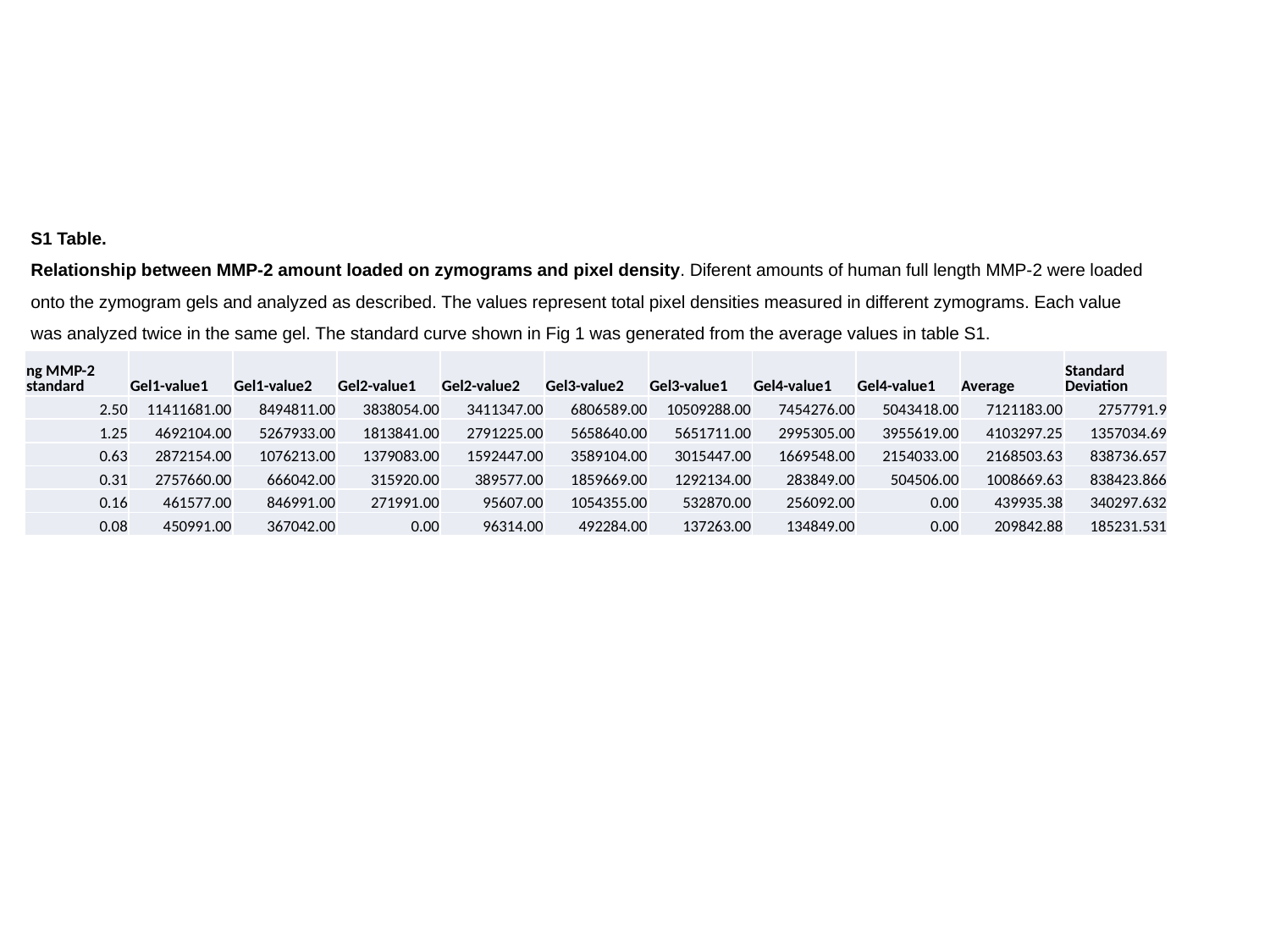

S1 Table.
Relationship between MMP-2 amount loaded on zymograms and pixel density. Diferent amounts of human full length MMP-2 were loaded onto the zymogram gels and analyzed as described. The values represent total pixel densities measured in different zymograms. Each value was analyzed twice in the same gel. The standard curve shown in Fig 1 was generated from the average values in table S1.
| ng MMP-2 standard | Gel1-value1 | Gel1-value2 | Gel2-value1 | Gel2-value2 | Gel3-value2 | Gel3-value1 | Gel4-value1 | Gel4-value1 | Average | Standard Deviation |
| --- | --- | --- | --- | --- | --- | --- | --- | --- | --- | --- |
| 2.50 | 11411681.00 | 8494811.00 | 3838054.00 | 3411347.00 | 6806589.00 | 10509288.00 | 7454276.00 | 5043418.00 | 7121183.00 | 2757791.9 |
| 1.25 | 4692104.00 | 5267933.00 | 1813841.00 | 2791225.00 | 5658640.00 | 5651711.00 | 2995305.00 | 3955619.00 | 4103297.25 | 1357034.69 |
| 0.63 | 2872154.00 | 1076213.00 | 1379083.00 | 1592447.00 | 3589104.00 | 3015447.00 | 1669548.00 | 2154033.00 | 2168503.63 | 838736.657 |
| 0.31 | 2757660.00 | 666042.00 | 315920.00 | 389577.00 | 1859669.00 | 1292134.00 | 283849.00 | 504506.00 | 1008669.63 | 838423.866 |
| 0.16 | 461577.00 | 846991.00 | 271991.00 | 95607.00 | 1054355.00 | 532870.00 | 256092.00 | 0.00 | 439935.38 | 340297.632 |
| 0.08 | 450991.00 | 367042.00 | 0.00 | 96314.00 | 492284.00 | 137263.00 | 134849.00 | 0.00 | 209842.88 | 185231.531 |
