# Supplementary material for: MMP-2 Isoforms in Aortic Tissue and Serum of Patients with Ascending Aortic Aneurysms and Aortic Root Aneurysms
Source: PLoS One. 2016 Nov 1;11(11):e0164308. doi: 10.1371/journal.pone.0164308 (PMC5089694; doi:10.1371/journal.pone.0164308)
Supplement: S2 Table — Protein extracts from ascending aortic tissue were diluted as indicated. Average pixel densitiy represents the mean of one sample run twice at in the same gel. StDev: Standard Deviation. (PPTX) [file pone.0164308.s005.pptx]

## Slide 1
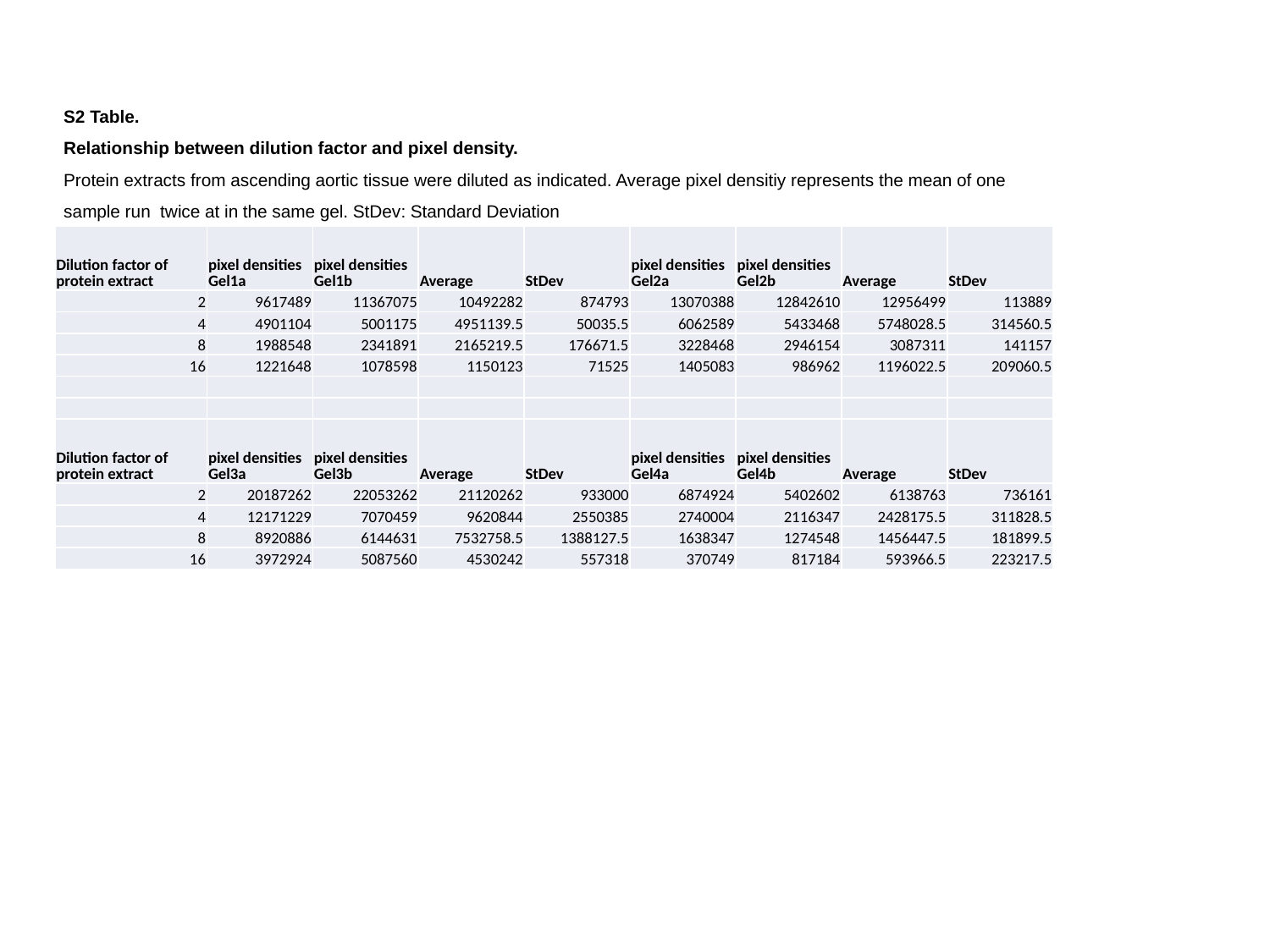

S2 Table.
Relationship between dilution factor and pixel density.
Protein extracts from ascending aortic tissue were diluted as indicated. Average pixel densitiy represents the mean of one sample run twice at in the same gel. StDev: Standard Deviation
| Dilution factor of protein extract | pixel densities Gel1a | pixel densities Gel1b | Average | StDev | pixel densities Gel2a | pixel densities Gel2b | Average | StDev |
| --- | --- | --- | --- | --- | --- | --- | --- | --- |
| 2 | 9617489 | 11367075 | 10492282 | 874793 | 13070388 | 12842610 | 12956499 | 113889 |
| 4 | 4901104 | 5001175 | 4951139.5 | 50035.5 | 6062589 | 5433468 | 5748028.5 | 314560.5 |
| 8 | 1988548 | 2341891 | 2165219.5 | 176671.5 | 3228468 | 2946154 | 3087311 | 141157 |
| 16 | 1221648 | 1078598 | 1150123 | 71525 | 1405083 | 986962 | 1196022.5 | 209060.5 |
| | | | | | | | | |
| | | | | | | | | |
| Dilution factor of protein extract | pixel densities Gel3a | pixel densities Gel3b | Average | StDev | pixel densities Gel4a | pixel densities Gel4b | Average | StDev |
| 2 | 20187262 | 22053262 | 21120262 | 933000 | 6874924 | 5402602 | 6138763 | 736161 |
| 4 | 12171229 | 7070459 | 9620844 | 2550385 | 2740004 | 2116347 | 2428175.5 | 311828.5 |
| 8 | 8920886 | 6144631 | 7532758.5 | 1388127.5 | 1638347 | 1274548 | 1456447.5 | 181899.5 |
| 16 | 3972924 | 5087560 | 4530242 | 557318 | 370749 | 817184 | 593966.5 | 223217.5 |
