# Supplementary material for: MMP-2 Isoforms in Aortic Tissue and Serum of Patients with Ascending Aortic Aneurysms and Aortic Root Aneurysms
Source: PLoS One. 2016 Nov 1;11(11):e0164308. doi: 10.1371/journal.pone.0164308 (PMC5089694; doi:10.1371/journal.pone.0164308)
Supplement: S3 Table — Serum MMP-2 was measured by Enzyme-linked Immunosorbent Assay. (PPTX) [file pone.0164308.s006.pptx]

## Slide 1
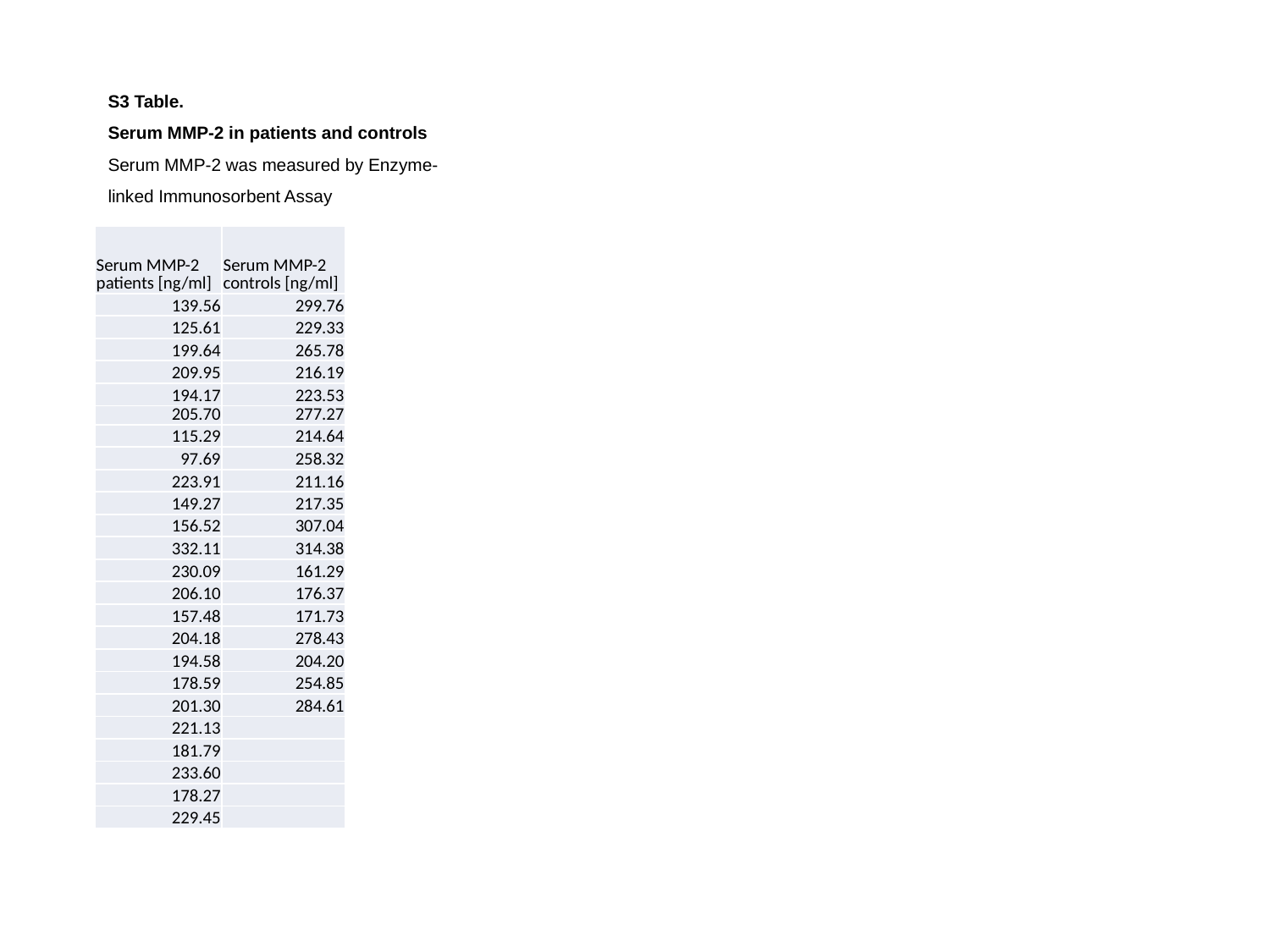

S3 Table.
Serum MMP-2 in patients and controls
Serum MMP-2 was measured by Enzyme-linked Immunosorbent Assay
| Serum MMP-2 patients [ng/ml] | Serum MMP-2 controls [ng/ml] |
| --- | --- |
| 139.56 | 299.76 |
| 125.61 | 229.33 |
| 199.64 | 265.78 |
| 209.95 | 216.19 |
| 194.17 | 223.53 |
| 205.70 | 277.27 |
| 115.29 | 214.64 |
| 97.69 | 258.32 |
| 223.91 | 211.16 |
| 149.27 | 217.35 |
| 156.52 | 307.04 |
| 332.11 | 314.38 |
| 230.09 | 161.29 |
| 206.10 | 176.37 |
| 157.48 | 171.73 |
| 204.18 | 278.43 |
| 194.58 | 204.20 |
| 178.59 | 254.85 |
| 201.30 | 284.61 |
| 221.13 | |
| 181.79 | |
| 233.60 | |
| 178.27 | |
| 229.45 | |
