# Supplementary material for: MMP-2 Isoforms in Aortic Tissue and Serum of Patients with Ascending Aortic Aneurysms and Aortic Root Aneurysms
Source: PLoS One. 2016 Nov 1;11(11):e0164308. doi: 10.1371/journal.pone.0164308 (PMC5089694; doi:10.1371/journal.pone.0164308)
Supplement: S4 Table — A: Experiment 1; B. Experiment 2; C: Experiment 3. (PPTX) [file pone.0164308.s007.pptx]

## Slide 1
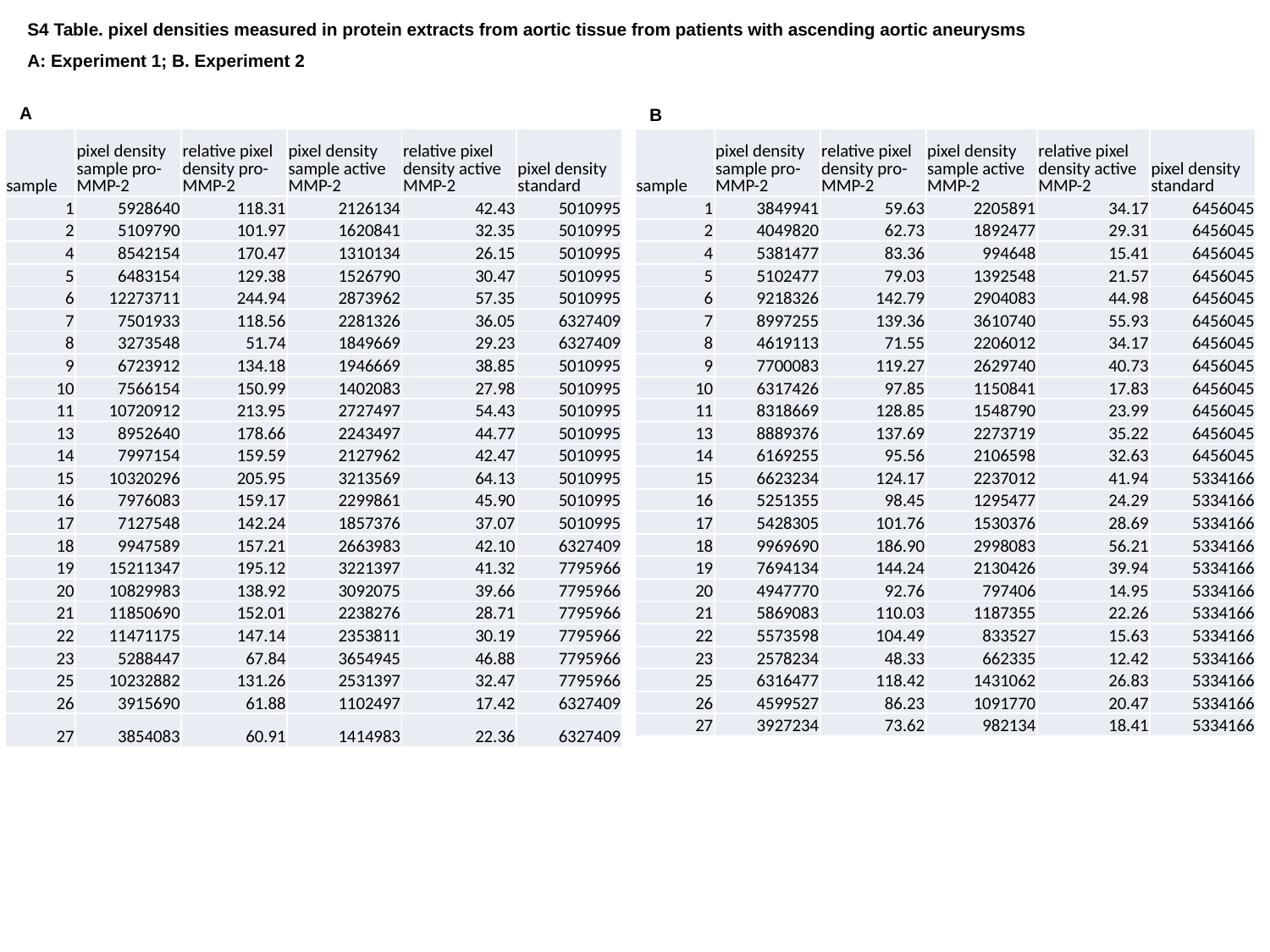

S4 Table. pixel densities measured in protein extracts from aortic tissue from patients with ascending aortic aneurysms
A: Experiment 1; B. Experiment 2
A
B
| sample | pixel density sample pro- MMP-2 | relative pixel density pro-MMP-2 | pixel density sample active MMP-2 | relative pixel density active MMP-2 | pixel density standard |
| --- | --- | --- | --- | --- | --- |
| 1 | 5928640 | 118.31 | 2126134 | 42.43 | 5010995 |
| 2 | 5109790 | 101.97 | 1620841 | 32.35 | 5010995 |
| 4 | 8542154 | 170.47 | 1310134 | 26.15 | 5010995 |
| 5 | 6483154 | 129.38 | 1526790 | 30.47 | 5010995 |
| 6 | 12273711 | 244.94 | 2873962 | 57.35 | 5010995 |
| 7 | 7501933 | 118.56 | 2281326 | 36.05 | 6327409 |
| 8 | 3273548 | 51.74 | 1849669 | 29.23 | 6327409 |
| 9 | 6723912 | 134.18 | 1946669 | 38.85 | 5010995 |
| 10 | 7566154 | 150.99 | 1402083 | 27.98 | 5010995 |
| 11 | 10720912 | 213.95 | 2727497 | 54.43 | 5010995 |
| 13 | 8952640 | 178.66 | 2243497 | 44.77 | 5010995 |
| 14 | 7997154 | 159.59 | 2127962 | 42.47 | 5010995 |
| 15 | 10320296 | 205.95 | 3213569 | 64.13 | 5010995 |
| 16 | 7976083 | 159.17 | 2299861 | 45.90 | 5010995 |
| 17 | 7127548 | 142.24 | 1857376 | 37.07 | 5010995 |
| 18 | 9947589 | 157.21 | 2663983 | 42.10 | 6327409 |
| 19 | 15211347 | 195.12 | 3221397 | 41.32 | 7795966 |
| 20 | 10829983 | 138.92 | 3092075 | 39.66 | 7795966 |
| 21 | 11850690 | 152.01 | 2238276 | 28.71 | 7795966 |
| 22 | 11471175 | 147.14 | 2353811 | 30.19 | 7795966 |
| 23 | 5288447 | 67.84 | 3654945 | 46.88 | 7795966 |
| 25 | 10232882 | 131.26 | 2531397 | 32.47 | 7795966 |
| 26 | 3915690 | 61.88 | 1102497 | 17.42 | 6327409 |
| 27 | 3854083 | 60.91 | 1414983 | 22.36 | 6327409 |
| sample | pixel density sample pro- MMP-2 | relative pixel density pro-MMP-2 | pixel density sample active MMP-2 | relative pixel density active MMP-2 | pixel density standard |
| --- | --- | --- | --- | --- | --- |
| 1 | 3849941 | 59.63 | 2205891 | 34.17 | 6456045 |
| 2 | 4049820 | 62.73 | 1892477 | 29.31 | 6456045 |
| 4 | 5381477 | 83.36 | 994648 | 15.41 | 6456045 |
| 5 | 5102477 | 79.03 | 1392548 | 21.57 | 6456045 |
| 6 | 9218326 | 142.79 | 2904083 | 44.98 | 6456045 |
| 7 | 8997255 | 139.36 | 3610740 | 55.93 | 6456045 |
| 8 | 4619113 | 71.55 | 2206012 | 34.17 | 6456045 |
| 9 | 7700083 | 119.27 | 2629740 | 40.73 | 6456045 |
| 10 | 6317426 | 97.85 | 1150841 | 17.83 | 6456045 |
| 11 | 8318669 | 128.85 | 1548790 | 23.99 | 6456045 |
| 13 | 8889376 | 137.69 | 2273719 | 35.22 | 6456045 |
| 14 | 6169255 | 95.56 | 2106598 | 32.63 | 6456045 |
| 15 | 6623234 | 124.17 | 2237012 | 41.94 | 5334166 |
| 16 | 5251355 | 98.45 | 1295477 | 24.29 | 5334166 |
| 17 | 5428305 | 101.76 | 1530376 | 28.69 | 5334166 |
| 18 | 9969690 | 186.90 | 2998083 | 56.21 | 5334166 |
| 19 | 7694134 | 144.24 | 2130426 | 39.94 | 5334166 |
| 20 | 4947770 | 92.76 | 797406 | 14.95 | 5334166 |
| 21 | 5869083 | 110.03 | 1187355 | 22.26 | 5334166 |
| 22 | 5573598 | 104.49 | 833527 | 15.63 | 5334166 |
| 23 | 2578234 | 48.33 | 662335 | 12.42 | 5334166 |
| 25 | 6316477 | 118.42 | 1431062 | 26.83 | 5334166 |
| 26 | 4599527 | 86.23 | 1091770 | 20.47 | 5334166 |
| 27 | 3927234 | 73.62 | 982134 | 18.41 | 5334166 |

## Slide 2
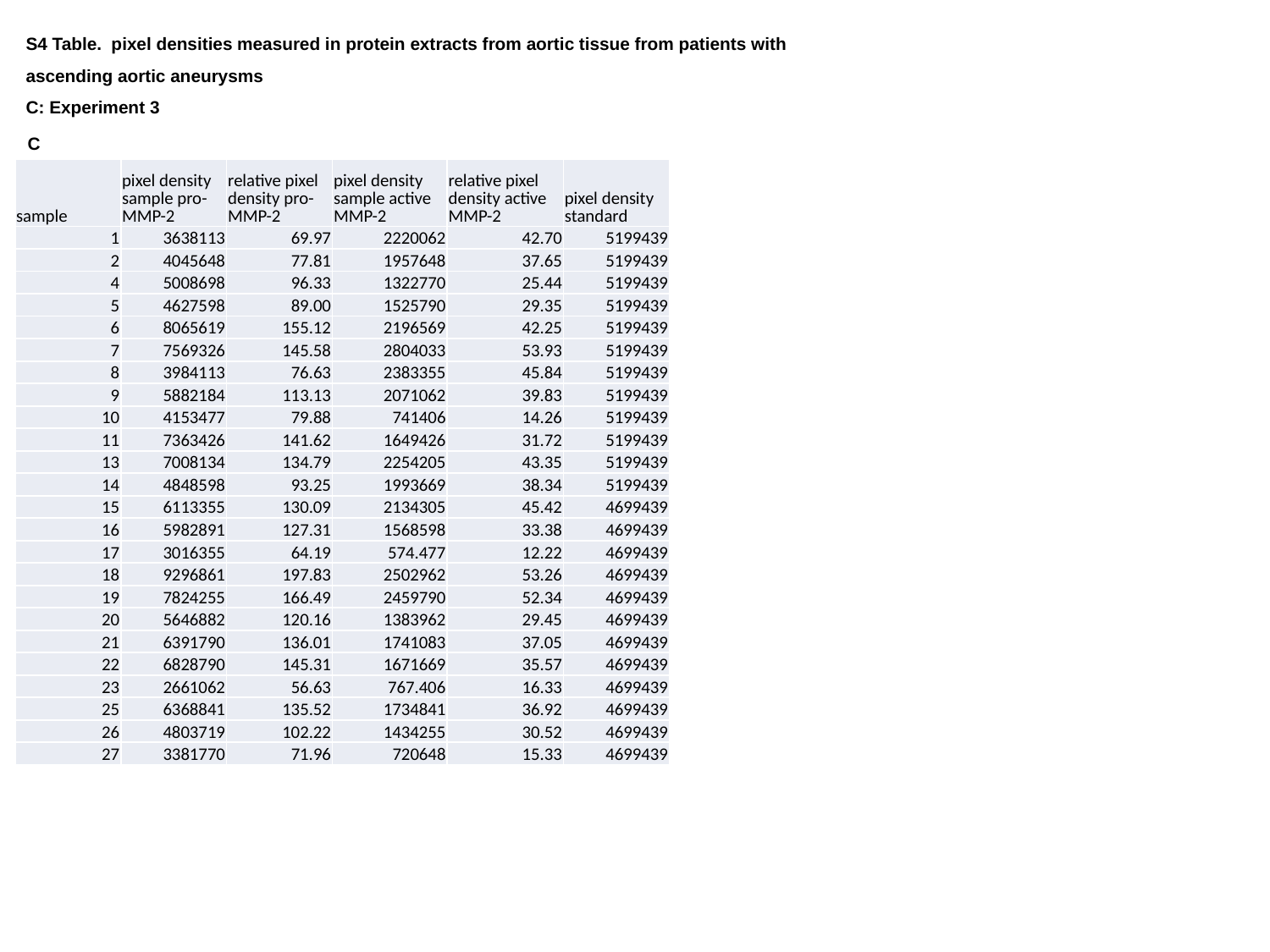

S4 Table. pixel densities measured in protein extracts from aortic tissue from patients with ascending aortic aneurysms
C: Experiment 3
C
| sample | pixel density sample pro- MMP-2 | relative pixel density pro-MMP-2 | pixel density sample active MMP-2 | relative pixel density active MMP-2 | pixel density standard |
| --- | --- | --- | --- | --- | --- |
| 1 | 3638113 | 69.97 | 2220062 | 42.70 | 5199439 |
| 2 | 4045648 | 77.81 | 1957648 | 37.65 | 5199439 |
| 4 | 5008698 | 96.33 | 1322770 | 25.44 | 5199439 |
| 5 | 4627598 | 89.00 | 1525790 | 29.35 | 5199439 |
| 6 | 8065619 | 155.12 | 2196569 | 42.25 | 5199439 |
| 7 | 7569326 | 145.58 | 2804033 | 53.93 | 5199439 |
| 8 | 3984113 | 76.63 | 2383355 | 45.84 | 5199439 |
| 9 | 5882184 | 113.13 | 2071062 | 39.83 | 5199439 |
| 10 | 4153477 | 79.88 | 741406 | 14.26 | 5199439 |
| 11 | 7363426 | 141.62 | 1649426 | 31.72 | 5199439 |
| 13 | 7008134 | 134.79 | 2254205 | 43.35 | 5199439 |
| 14 | 4848598 | 93.25 | 1993669 | 38.34 | 5199439 |
| 15 | 6113355 | 130.09 | 2134305 | 45.42 | 4699439 |
| 16 | 5982891 | 127.31 | 1568598 | 33.38 | 4699439 |
| 17 | 3016355 | 64.19 | 574.477 | 12.22 | 4699439 |
| 18 | 9296861 | 197.83 | 2502962 | 53.26 | 4699439 |
| 19 | 7824255 | 166.49 | 2459790 | 52.34 | 4699439 |
| 20 | 5646882 | 120.16 | 1383962 | 29.45 | 4699439 |
| 21 | 6391790 | 136.01 | 1741083 | 37.05 | 4699439 |
| 22 | 6828790 | 145.31 | 1671669 | 35.57 | 4699439 |
| 23 | 2661062 | 56.63 | 767.406 | 16.33 | 4699439 |
| 25 | 6368841 | 135.52 | 1734841 | 36.92 | 4699439 |
| 26 | 4803719 | 102.22 | 1434255 | 30.52 | 4699439 |
| 27 | 3381770 | 71.96 | 720648 | 15.33 | 4699439 |
