# Supplementary material for: MMP-2 Isoforms in Aortic Tissue and Serum of Patients with Ascending Aortic Aneurysms and Aortic Root Aneurysms
Source: PLoS One. 2016 Nov 1;11(11):e0164308. doi: 10.1371/journal.pone.0164308 (PMC5089694; doi:10.1371/journal.pone.0164308)
Supplement: S5 Table — Each standard value represents 0.33 ng human full length MMP-2. (PPTX) [file pone.0164308.s008.pptx]

## Slide 1
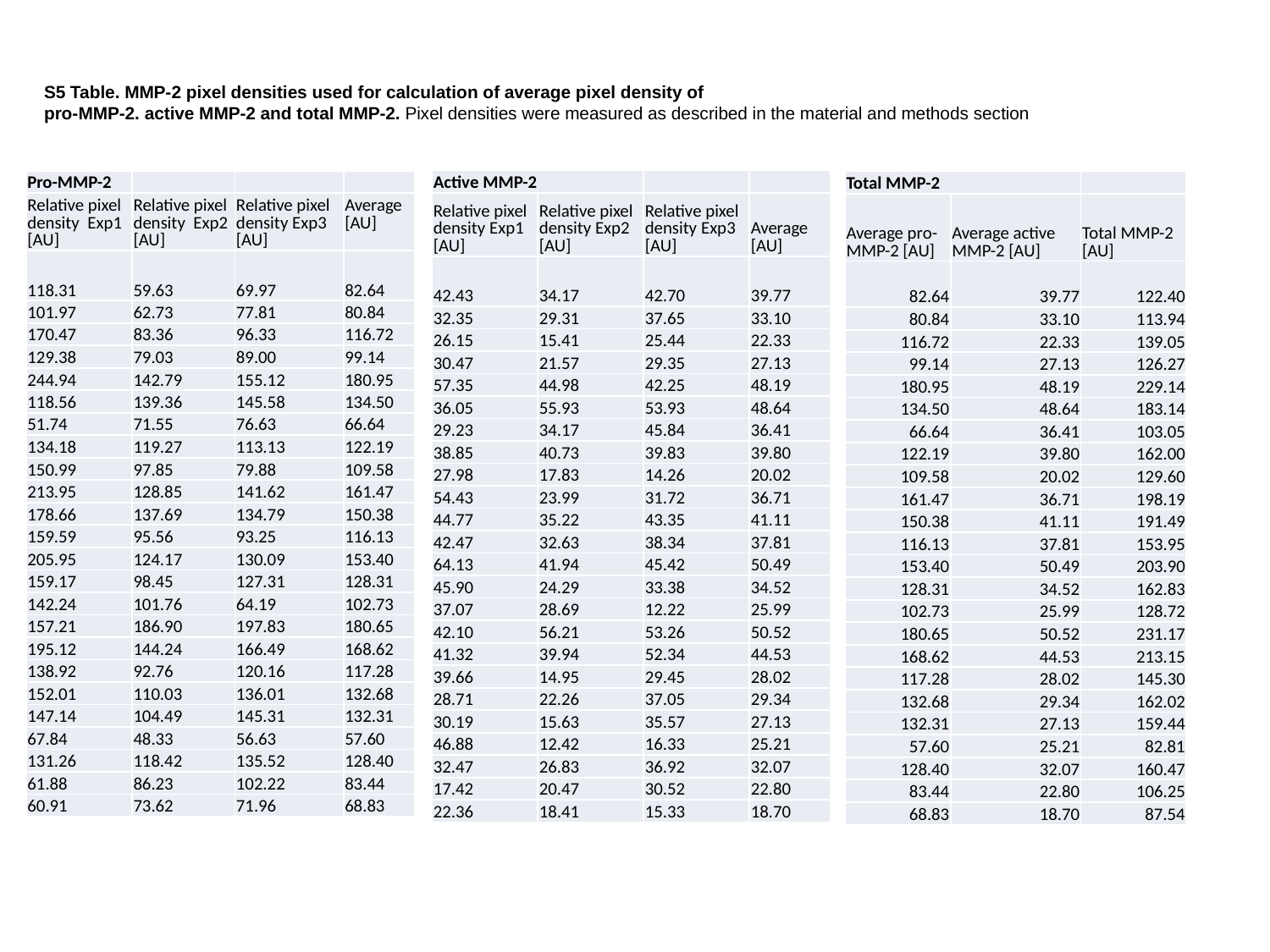

S5 Table. MMP-2 pixel densities used for calculation of average pixel density of
pro-MMP-2. active MMP-2 and total MMP-2. Pixel densities were measured as described in the material and methods section
| Active MMP-2 | | | |
| --- | --- | --- | --- |
| Relative pixel density Exp1 [AU] | Relative pixel density Exp2 [AU] | Relative pixel density Exp3 [AU] | Average [AU] |
| 42.43 | 34.17 | 42.70 | 39.77 |
| 32.35 | 29.31 | 37.65 | 33.10 |
| 26.15 | 15.41 | 25.44 | 22.33 |
| 30.47 | 21.57 | 29.35 | 27.13 |
| 57.35 | 44.98 | 42.25 | 48.19 |
| 36.05 | 55.93 | 53.93 | 48.64 |
| 29.23 | 34.17 | 45.84 | 36.41 |
| 38.85 | 40.73 | 39.83 | 39.80 |
| 27.98 | 17.83 | 14.26 | 20.02 |
| 54.43 | 23.99 | 31.72 | 36.71 |
| 44.77 | 35.22 | 43.35 | 41.11 |
| 42.47 | 32.63 | 38.34 | 37.81 |
| 64.13 | 41.94 | 45.42 | 50.49 |
| 45.90 | 24.29 | 33.38 | 34.52 |
| 37.07 | 28.69 | 12.22 | 25.99 |
| 42.10 | 56.21 | 53.26 | 50.52 |
| 41.32 | 39.94 | 52.34 | 44.53 |
| 39.66 | 14.95 | 29.45 | 28.02 |
| 28.71 | 22.26 | 37.05 | 29.34 |
| 30.19 | 15.63 | 35.57 | 27.13 |
| 46.88 | 12.42 | 16.33 | 25.21 |
| 32.47 | 26.83 | 36.92 | 32.07 |
| 17.42 | 20.47 | 30.52 | 22.80 |
| 22.36 | 18.41 | 15.33 | 18.70 |
| Pro-MMP-2 | | | |
| --- | --- | --- | --- |
| Relative pixel density Exp1 [AU] | Relative pixel density Exp2 [AU] | Relative pixel density Exp3 [AU] | Average [AU] |
| 118.31 | 59.63 | 69.97 | 82.64 |
| 101.97 | 62.73 | 77.81 | 80.84 |
| 170.47 | 83.36 | 96.33 | 116.72 |
| 129.38 | 79.03 | 89.00 | 99.14 |
| 244.94 | 142.79 | 155.12 | 180.95 |
| 118.56 | 139.36 | 145.58 | 134.50 |
| 51.74 | 71.55 | 76.63 | 66.64 |
| 134.18 | 119.27 | 113.13 | 122.19 |
| 150.99 | 97.85 | 79.88 | 109.58 |
| 213.95 | 128.85 | 141.62 | 161.47 |
| 178.66 | 137.69 | 134.79 | 150.38 |
| 159.59 | 95.56 | 93.25 | 116.13 |
| 205.95 | 124.17 | 130.09 | 153.40 |
| 159.17 | 98.45 | 127.31 | 128.31 |
| 142.24 | 101.76 | 64.19 | 102.73 |
| 157.21 | 186.90 | 197.83 | 180.65 |
| 195.12 | 144.24 | 166.49 | 168.62 |
| 138.92 | 92.76 | 120.16 | 117.28 |
| 152.01 | 110.03 | 136.01 | 132.68 |
| 147.14 | 104.49 | 145.31 | 132.31 |
| 67.84 | 48.33 | 56.63 | 57.60 |
| 131.26 | 118.42 | 135.52 | 128.40 |
| 61.88 | 86.23 | 102.22 | 83.44 |
| 60.91 | 73.62 | 71.96 | 68.83 |
| Total MMP-2 | | |
| --- | --- | --- |
| Average pro-MMP-2 [AU] | Average active MMP-2 [AU] | Total MMP-2 [AU] |
| 82.64 | 39.77 | 122.40 |
| 80.84 | 33.10 | 113.94 |
| 116.72 | 22.33 | 139.05 |
| 99.14 | 27.13 | 126.27 |
| 180.95 | 48.19 | 229.14 |
| 134.50 | 48.64 | 183.14 |
| 66.64 | 36.41 | 103.05 |
| 122.19 | 39.80 | 162.00 |
| 109.58 | 20.02 | 129.60 |
| 161.47 | 36.71 | 198.19 |
| 150.38 | 41.11 | 191.49 |
| 116.13 | 37.81 | 153.95 |
| 153.40 | 50.49 | 203.90 |
| 128.31 | 34.52 | 162.83 |
| 102.73 | 25.99 | 128.72 |
| 180.65 | 50.52 | 231.17 |
| 168.62 | 44.53 | 213.15 |
| 117.28 | 28.02 | 145.30 |
| 132.68 | 29.34 | 162.02 |
| 132.31 | 27.13 | 159.44 |
| 57.60 | 25.21 | 82.81 |
| 128.40 | 32.07 | 160.47 |
| 83.44 | 22.80 | 106.25 |
| 68.83 | 18.70 | 87.54 |
