# Supplementary material for: MMP-2 Isoforms in Aortic Tissue and Serum of Patients with Ascending Aortic Aneurysms and Aortic Root Aneurysms
Source: PLoS One. 2016 Nov 1;11(11):e0164308. doi: 10.1371/journal.pone.0164308 (PMC5089694; doi:10.1371/journal.pone.0164308)
Supplement: S6 Table — MMP-2 values from zymograms were calculated as described in Material and methods. (PPTX) [file pone.0164308.s009.pptx]

## Slide 1
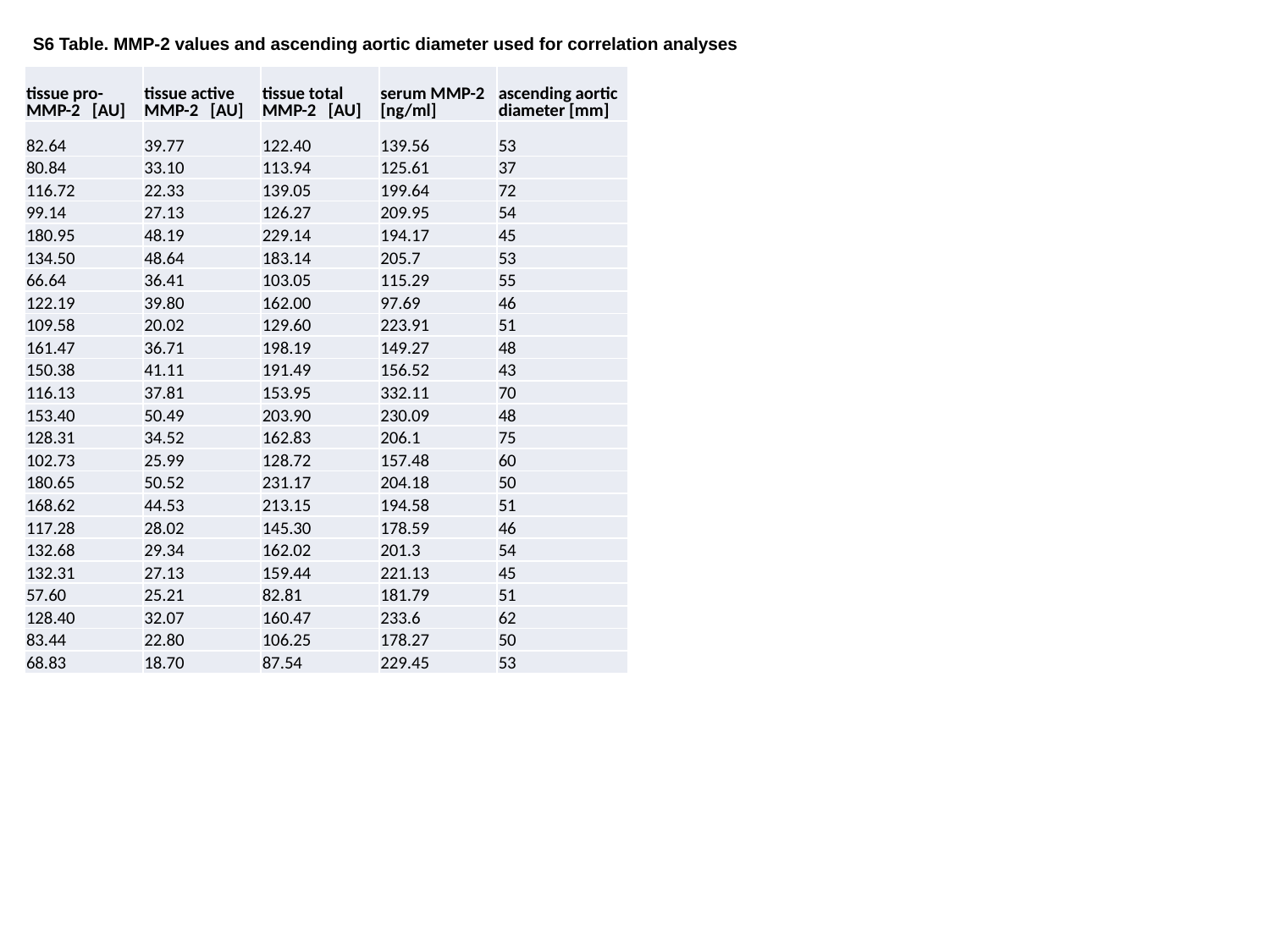

S6 Table. MMP-2 values and ascending aortic diameter used for correlation analyses
| tissue pro-MMP-2 [AU] | tissue active MMP-2 [AU] | tissue total MMP-2 [AU] | serum MMP-2 [ng/ml] | ascending aortic diameter [mm] |
| --- | --- | --- | --- | --- |
| 82.64 | 39.77 | 122.40 | 139.56 | 53 |
| 80.84 | 33.10 | 113.94 | 125.61 | 37 |
| 116.72 | 22.33 | 139.05 | 199.64 | 72 |
| 99.14 | 27.13 | 126.27 | 209.95 | 54 |
| 180.95 | 48.19 | 229.14 | 194.17 | 45 |
| 134.50 | 48.64 | 183.14 | 205.7 | 53 |
| 66.64 | 36.41 | 103.05 | 115.29 | 55 |
| 122.19 | 39.80 | 162.00 | 97.69 | 46 |
| 109.58 | 20.02 | 129.60 | 223.91 | 51 |
| 161.47 | 36.71 | 198.19 | 149.27 | 48 |
| 150.38 | 41.11 | 191.49 | 156.52 | 43 |
| 116.13 | 37.81 | 153.95 | 332.11 | 70 |
| 153.40 | 50.49 | 203.90 | 230.09 | 48 |
| 128.31 | 34.52 | 162.83 | 206.1 | 75 |
| 102.73 | 25.99 | 128.72 | 157.48 | 60 |
| 180.65 | 50.52 | 231.17 | 204.18 | 50 |
| 168.62 | 44.53 | 213.15 | 194.58 | 51 |
| 117.28 | 28.02 | 145.30 | 178.59 | 46 |
| 132.68 | 29.34 | 162.02 | 201.3 | 54 |
| 132.31 | 27.13 | 159.44 | 221.13 | 45 |
| 57.60 | 25.21 | 82.81 | 181.79 | 51 |
| 128.40 | 32.07 | 160.47 | 233.6 | 62 |
| 83.44 | 22.80 | 106.25 | 178.27 | 50 |
| 68.83 | 18.70 | 87.54 | 229.45 | 53 |
